# Osteoblast-derived osteomodulin restrains osteoclastogenesis via ITGB8/RRM2-mediated reduction of mitochondrial respiration and mitochondrial ATP production

**DOI:** 10.1038/s12276-026-01682-7

**Published:** 2026-03-12

**Authors:** Xiaowen Jiang, Han Chen, Weiduo Hou, Chengxin Dai, Ruijie Zhang, Erman Chen, Jingjing Zhang, Wanyi Wu, Jianbin Xu, Mo Chen, Liang Liu, Xun Tan, Xiaoxiao Ji, Xiaoyong Wu, Kanbin Wang, Chaorong Yu, Xiaohua Yu, Weixu Li, Wei Zhang

**Affiliations:** 1 Department of Orthopedic Surgery of the Second Affiliated Hospital, School of Medicine, Zhejiang University, Hangzhou, China; 2 Orthopedics Research, Institute of Zhejiang University, Hangzhou, China; 3 Key Laboratory of Motor System Disease Research and Precision Therapy of Zhejiang Province, Hangzhou City, China; 4 Taizhou Hospital of Zhejiang Province affiliated to Wenzhou Medical University, Wenzhou, China; 5 College of Pharmacy, Hangzhou Normal University, Hangzhou, China; 6 Department of Rheumatology, Second Affiliated Hospital, School of Medicine, Zhejiang University, Hangzhou, China; 7 Department of Neurology, Second Affiliated Hospital, College of Medicine, Zhejiang University, Hangzhou, China

**Keywords:** Cell biology, Diseases

## Abstract

Osteoporosis is driven in part by excessive osteoclast-mediated bone resorption, yet osteoblast-derived extracellular cues that restrain osteoclast bioenergetics remain incompletely defined. Here we identify osteomodulin (OMD), a matrix-associated osteoblast-derived protein that is reduced in the bone tissue and serum of postmenopausal patients with osteoporosis. Inducible global or osteoblast-specific *Omd* deletion exacerbates bone loss and increases osteoclast activity, whereas osteoclast precursor-specific deletion produces no overt skeletal phenotype. Mechanistically, OMD engages integrin β8 on osteoclast precursors, suppresses RhoA activity and enhances YAP phosphorylation, thereby reducing YAP/TEAD occupancy at the ribonucleotide reductase M2 (RRM2) promoter and repressing *Rrm2* transcription. Consistent with RRM2’s role in maintaining the dNTP pools required for mitochondrial DNA replication, OMD decreases mtDNA copy number and the abundance of electron transport chain proteins, leading to reduced mitochondrial respiration and ATP production, with only limited glycolytic compensation. Finally, recombinant OMD supplementation or pharmacologic RRM2 inhibition mitigates ovariectomy and lipopolysaccharide-induced bone loss. Together, our findings identify an OMD-integrin β8-RhoA-YAP/TEAD-RRM2 axis that links extracellular matrix signaling to mitochondrial respiration and mitochondrial ATP production during osteoclastogenesis.

## Osteomodulin links bone loss to mitochondrial function

Osteoporosis is a condition characterized by reduced bone strength, increasing the risk of fractures, particularly in older adults and postmenopausal women. This study explores a protein called osteomodulin (OMD) and its role in bone health. Researchers found that OMD levels are lower in people with osteoporosis. They used mice to study how OMD affects bone cells. By removing OMD in mice, they observed increased bone loss and more active bone-resorbing cells called osteoclasts. They also discovered that OMD interacts with a receptor called ITGB8 on osteoclast precursors, reducing their activity by affecting energy production in the cells. The study suggests that OMD helps maintain bone health by regulating osteoclast activity. The researchers propose that increasing OMD levels or targeting related pathways could be a new way to treat osteoporosis.

This summary was initially drafted using artificial intelligence, then revised and fact-checked by the author.

## Introduction

Osteoporosis, a condition characterized by decreased bone density and increased fracture risk, affects the aging population disproportionately, particularly postmenopausal women^1^. Despite the existence of pharmacological interventions, many of these drugs are constrained by their single-target action, significant toxicity and adverse side effects, particularly in the context of osteoporosis treatment for the elderly, who often have comorbid cardiovascular diseases^1^. Better therapies are needed; thus, a comprehensive understanding of the underlying pathological mechanisms of osteoporosis is desirable^2^.

Bone homeostasis is maintained through a delicate balance between bone resorption and bone formation, controlled by osteoblasts and osteoclasts, respectively^3^. Therefore, the interactions between osteoblasts and osteoclasts are key to maintaining bone structure and function^4^. In this process, bone-specific osteokines (BOKs) play a pivotal regulatory role. Defined as signaling molecules secreted by bone-resident cells—including osteoblasts, osteoclasts and bone marrow mesenchymal stem cells—these cytokines orchestrate the dynamic equilibrium of bone metabolism by modulating skeletal formation, resorption and repair processes^5^. Hence, BOKs are regarded as key mediators of intercellular signaling, responsible for conveying information among various types of bone cell and regulating their survival, differentiation and function^6^. For instance, osteoblasts secrete key regulatory proteins, such as receptor-activated NF-κB ligand (RANKL) and osteoprotegerin, both of which are crucial for osteoclastogenesis^7,8^. Osteoclasts can also release factors that regulate osteoblast function, such as sphingosine 1 phosphate, collagen triple helix repeat containing 1 and semaphorin 4D^7,8^. These BOKs are not only crucial for maintaining skeletal health but also implicated in the pathogenesis of bone-related diseases, such as fractures, osteonecrosis and inflammation^9,10^. Although research on the mechanisms of intercellular signaling and the regulation of the bone microenvironment has made some progress recent years, there is still a lack of clinically feasible intervention strategies for precisely targeting osteoclast differentiation at the cellular level and effectively slowing down bone loss.

Osteomodulin (OMD), a member of the osteokine group, is classified within the small leucine-rich proteoglycan (SLRP) family^11^. SLRP family proteins are important bioactive components of the extracellular matrix (ECM)^12^ and play critical roles in maintaining homeostasis of the bone tissue microenvironment under various conditions, including osteoclast formation^13,14^ and osteoblast differentiation^15–17^. The role of OMD in the broader human microenvironment has attracted much attention^18^, and studies have revealed that OMD may participate in the inflammatory response^19,20^. Furthermore, both pro-inflammatory and pro-osteogenic stimuli have been found to induce OMD localization in calcified cardiovascular tissues, specifically in the medial and intimal α-SMA regions, where OMD appears to mediate smooth muscle cell calcification^21^. OMD mRNA levels also correlate with the stability of human atherosclerotic plaques^21,22^. Predominantly expressed in mature osteoblasts, OMD is thought to regulate mineralization^23^. Notably, osteoblast differentiation upregulates OMD expression, and OMD overexpression promotes osteoblasts maturation in vitro^17,23^. Its interaction with the bone microenvironment underscores OMD’s multifaceted role in bone health, influencing cellular behavior and interactions within the bone niche^18,24^. However, despite these advances, the regulation of OMD in osteoporosis and its functional relevance to osteoclast-driven skeletal pathology remain poorly characterized.

In this study, we identified an association between OMD and osteoporosis by analyzing various osteoporotic datasets. Clinical samples from patients further confirmed the negative correlation between OMD levels and postmenopausal bone loss. Reduced bone mass and excessive osteoclast activation have been observed in global OMD-knockout (gKO) mice, and similar findings have been noted in osteoblast-specific knockout models. Mechanistically, OMD engages integrin β8 to inhibit ras homolog family member A (RhoA)-Yes1 associated transcriptional regulator (YAP) signaling, leading to reduced YAP/TEA domain transcription factor (TEAD) occupancy at the *Rrm2* promoter and compromised mitochondrial respiration and mitochondrial ATP in osteoclast precursors. Importantly, treatment with either OMD or an RRM2 inhibitor in mice models induced by ovariectomy (OVX) and lipopolysaccharide (LPS) partly mitigated bone loss. In summary, our findings underscore the critical role of osteoblast-derived OMD in the regulation of osteoclast formation and the maintenance of bone homeostasis.

## Materials and methods

### Study approval

This study was conducted in accordance with the principles of the Basel Declaration and the recommendations of Zhejiang University. All in vivo animal experiments and protocols were approved by the Animal Ethics Committee of the Zhejiang University (no. 2021147). Following the ARRIVE ((Animal Research: Reporting of In Vivo Experiments) guidelines and the National Research Council’s Guide for the Care and Use of Laboratory Animals, this study ensured the ethical and humane treatment of animals. The approval date was 21 July 2021.

### Human samples

The collection of human bone tissue and serum samples was approved by the Ethics Committee of the Second Affiliated Hospital of School Medicine, Zhejiang University (no. 20210410), and the methods adhered to the guidelines of the Declaration of Helsinki. Written informed consent was obtained from all participants. Descriptive characteristics of the human samples are listed in Supplementary Table 1.

### Mice

*Omd*^flox^ mice with a C57BL/6 background, as well as CAG-ERT2-cre, LysM-cre and OC-cre mice, were purchased from Cyagen Biological Information Technology. DMP1-cre transgenic mice were a gift from Dr. Mengrui Wu. The flox sequences flanking exon 3 of the *Omd* gene facilitate Cre recombinase-mediated excision, enabling targeted gene manipulation. *Omd*^flox^ mice were subsequently crossed with CAG-ERT2-cre, LysM-cre, OC-cre and DMP1-cre mice to generate OMD-knockout mice. Tamoxifen (T5648, Sigma-Aldrich) was dissolved in corn oil containing 2% ethanol. At 8 weeks of age, the *Omd*^flox^ and *Omd*^ΔCAG^ mice were administered tamoxifen (1 mg per dose, twice daily for 5 consecutive days) or vehicle control to induce global *Omd* deletion.

### Reagents

α-MEM and fetal bovine serum (FBS) were purchased from Amizona Scientific LLC. Recombinant mouse macrophage colony-stimulating factor (M-CSF) (AM10003-050; AMIZONA) and RANKL (AM10004-050; AMIZONA) were used. Osteoclasts were stained using the tartrate-resistant acid phosphatase (TRAP) staining kit (AMK1005-150; AMIZONA). Cell Counting Kit-8 (CCK-8) reagent was purchased from Boyce (C0037). Osalmid (HY-B2116; MCE), SB-431542 (HY-10431; MCE), TRULI (HY-138489; MCE), recombinant human OMD protein (2884-AD; R&D Systems), Human Osteoadherin/OMD ELISA Kit (EK2017; Boster Biological Technology), Mouse Osteoadherin/OMD ELISA Kit (EK2018; Boster Biological Technology), Mouse β-CTX (Beta Crosslaps) ELISA Kit (E-EL-M0372; Elabscience), RhoA G-LISA Activation Assay Kit (BK124, Cytoskeleton), Mouse/Rat TGF-β ELISA Kit (PT878; Beyotime), 2-NBDG (N13195; Thermo Fisher), phosphate-buffered saline (PBS) (CR-10010; Cienry), collagenase II (C2-BIOC; Sigma), penicillin-streptomycin (100×) (HY-K1006; MCE), trypsin (25300062; Gibco) and 1,25-dihydroxyvitamin D3 (HY-10002; MCE) were used. Small interfering RNA (siRNA) was purchased from GenePharma. Adenovirus-mediated *Rrm2* was purchased from GENECHEM.

### Cell transfection and stable cell construction

To knock down or overexpress target proteins, we used RNAiMAX (13778150; Thermo Fisher) and siRNA to transfect bone marrow-derived macrophages (BMMs). Lipofectamine 3000 (L3000008; Thermo Fisher) and tagged plasmids were used to transfect HEK-293T (CL-0005; Procell) and RAW 264.7 cells (CL-0190; Procell).

### BMM isolation and osteoclast differentiation

BMMs were isolated from C57BL/6 mice and cultured in α-MEM supplemented with 10% FBS. Osteoclast differentiation was induced by treatment with M-CSF (30 ng/ml) and RANKL (100 ng/ml), or M-CSF (30 ng/ml) and LPS (100 ng/ml). Osteoclast formation was assessed using TRAP staining. TRAP-positive multinucleated cells (≥3 nuclei) were counted as osteoclasts.

### Osteoblast isolation and culture

Osteoblasts were isolated from the calvaria of 6-week-old male C57BL/6J mice as described previously^25^. In brief, mice were euthanized via intraperitoneal injection of a lethal dose of sodium pentobarbital. The calvariae were carefully dissected and cleared of soft tissue under a stereomicroscope, then placed in Petri dishes containing PBS. Cleaned calvariae were cut into 1–2-mm² pieces and washed thoroughly with PBS. The bone fragments were then incubated in a 2 mg/ml solution of collagenase II for 2 h at 37 °C in a shaking water bath to remove any remaining soft tissue and adhering cells. Following incubation, the fragments were washed with α-MEM, supplemented with 10% FBS. The fragments were then cultured in α-MEM supplemented with 10% FBS and 1% antibiotics in 25-cm² culture flasks. The cultures were maintained in a humidified incubator at 37 °C with 5% CO₂. Upon reaching confluence, the bone fragments were removed, and the confluent cell layers were trypsinized and replated. All cells were passaged for up to three generations.

### Coculture of osteoblasts and osteoclast precursors

Osteoblasts isolated from the calvaria were seeded onto the upper chamber of 96-well (8 × 10³ cells per well) or 12-well (7 × 10⁴ cells per well) transwell inserts as described previously^25^. After 24 h, osteoclast precursors (2 × 10⁵ or 2 × 10^6^ cells per well) were seeded onto the lower chamber of the transwell system. The cocultures were maintained for 3 or 9 days. Cultures were prepared using α-MEM supplemented with 10 nM vitamin D3, with the medium refreshed every 3 days.

### Genotype analysis using agarose gel electrophoresis

Approximately 3 weeks after birth, a small tissue segment was collected from the tails of neonatal mice. Genomic DNA extracted from the tail was analyzed using polymerase chain reaction (PCR) to determine the genotype of the mice. The floxed *Omd* alleles CAG-cre, LysM-cre, OC-cre and DMP1-cre were identified using the primers listed in Supplementary Table 2.

### Histology

Mouse femurs were decalcified in 12% EDTA-2Na at room temperature for approximately 2 weeks and then embedded in paraffin. Sections were subjected to TRAP and hematoxylin and eosin (H&E) staining. The number of osteoclasts per bone surface (N.Oc/BS) and the number of osteoclasts covering each bone surface (Oc.S/BS) were observed under a microscope (Leica).

### Micro-CT analysis

For micro-computed tomography (micro-CT) analysis, the distal femurs of appropriately aged mice were scanned using a μ-CT scanner (Skyscan 1072) with an X-ray energy of 80 μA/70 kV and an isotropic resolution of 9 μm. Trabecular bone was analyzed in 100 consecutive slices distal to the growth plate. Parameters such as bone volume per total volume (BV/TV), trabecular thickness (Tb.Th), trabecular separation (Tb.Sp) and trabecular number (Tb.N) were analyzed using CT assessment software as previously described^26^.

### CCK-8 assay

To evaluate the cytotoxic effects of OMD and osalmid, BMMs were plated in 96-well plates (8 × 10³ cells per well) and treated with OMD or osalmid for 48 h. Subsequently, BMMs were incubated with 10% CCK-8 solution (C0037; Beyotime) at 37 °C for 1 h. Absorbance at 450 nm was measured using a microplate reader.

### F-actin ring formation assay

The effect of OMD on F-actin ring formation was analyzed using phalloidin (C2201S; Beyotime). BMMs were seeded into 96-well plates (8 × 10³ cells per well) and incubated with 100 ng/ml of OMD and fresh osteoclastogenesis induction medium for either 2 or 4 days. Subsequently, cells were fixed with 4% paraformaldehyde (Sigma-Aldrich) and permeabilized with 0.1% Triton X-100. Cells were then stained with rhodamine-conjugated phalloidin in 2% bovine serum albumin (1:200) for 1 h. Nuclei were visualized by staining with 4′,6-diamidino-2-phenylindole for 5 min. Fluorescent images of F-actin were acquired using a fluorescence microscope (Leica). Three fields were randomly captured, and ImageJ software (NIH) was used to quantify the size of the F-actin rings.

### Quantitative real-time PCR (qRT–PCR)

Following treatment, the cells were lysed. Total RNA was extracted as previously described^27^. Real-time PCR analysis was performed using the SYBR Green PCR Kit (TaKaRa) on the ABI StepOnePlus System (Applied Biosystems). The cycling conditions were as described previously^28^. Primers were designed and synthesized by Sangon Biotech. β-Actin was used as the housekeeping gene, and gene expression levels were calculated using the 2^−ΔΔCt^ method. The primer sequences are listed in Supplementary Table 2.

### ChIP and quantitative PCR

Approximately 5 × 10^7^ BMMs cells were stimulated by M-CSF (30 ng/ml) and RANKL (100 ng/ml) with or without OMD (100 ng/ml). Chromatin immunoprecipitation (ChIP) assays were performed using the Pierce Magnetic ChIP Kit (26157, Thermo Fisher). Immunoprecipitation was performed with anti-TEAD1 antibodies (ABclonal, A13366). Primers involved in qRT–PCR are listed in Supplementary Table 2, which are designed on the basis of the known TEAD binding region of the *Rrm2* promoter^29^.

### WB analysis

BMMs (3 × 10^5^ cells per well) were seeded in six-well plates. Cells were cultured in the presence of 30 ng/ml M-CSF, with or without 100 ng/ml RANKL. Protein extraction in each well of the plate was performed using 200 μl of RIPA buffer (Solar Bio) supplemented with 100× phosphatase inhibitor cocktail (CWBIO), 100 mM phenylmethanesulfonyl fluoride (ST2573-5g, Beyotime) and protease inhibitor cocktail (Millipore). The lysate was centrifuged at 12,000 rpm for 15 min, and the supernatant was heated at 100 °C for 10 min. Sodium dodecyl sulfate–polyacrylamide gel electrophoresis was performed according to a previously established protocol^30^. Protein bands were visualized using the Amersham Imager 600 (GE) and quantified using ImageJ. Western blotting (WB) was performed with the following antibodies: anti-GAPDH (1:10,000, 60004-1-IG; Proteintech), anti-β-actin (1:10,000, 66009-1-IG; Proteintech), anti-cathepsin K (CTSK) (1:100, sc-48353; Santa Cruz), anti-NFATC1 (1:100, sc-7294; Santa Cruz), anti-matrix metallopeptidase 9 (MMP9) (1:1,000, 10375-2-AP; Proteintech), anti-RRM2 (1:100, sc-81850; Santa Cruz), anti-ITGB8 (1:1,000, 29775-1-AP; Proteintech), anti-OMD (1:1,000, ab154249; Abcam), anti-RUNX2 (1:1,000, 20700-1-AP; Proteintech), anti-collagen type I (1:1,000, 14695-1-AP; Proteintech), anti-ATP Synthase F1 Subunit Alpha (ATP5A1) (1:10,000, 14676-1-AP; Proteintech), anti-ubiquinol-cytochrome c reductase core protein 2 (UQCRC2) (1:10,000, 67547-1-Ig; Proteintech), anti-succinate dehydrogenase subunits B (SDHB) (1:10,000, 10620-1-AP; Proteintech), anti-cytochrome oxidase subunit IV (MTCO4) (1:1,000, 11242-1-AP; Proteintech), anti-NADH-ubiquinone oxidoreductase ASHI subunit (NDUFB8) (1:10,000, 14794-1-AP; Proteintech), anti-FLAG (1:10,000, 20543-1-AP; Proteintech), anti-HA (1:10,000, 51064-2-AP; Proteintech), anti-YAP (1:1,000, ET1608-30; HUABIO), anti-p-YAP (S127) (1:1,000, ET1611–69; HUABIO), anti-Smad2 (1:1,000, ET1604-22; HUABIO), anti-p-Smad2 (1:5,000, ET1612-32; HUABIO), anti-Smad3 (1:5,000, ET1607-41; HUABIO), anti-p-Smad3 (1:2,000, ET1609-41; HUABIO), anti-AKT (1:1,000, #9272, Cell Signaling Technology), anti-p-AKT (1:2,000, #4060, Cell Signaling Technology), anti-ERK (1:1,000, #9102, Cell Signaling Technology), anti-p-ERK (1:1,000, #4695, Cell Signaling Technology), anti-p38 (1:1,000, #9212, Cell Signaling Technology), anti-p-p38 (1:1,000, #4511, Cell Signaling Technology), anti-JNK (1:5,000, 51153-1-AP; Proteintech) and anti-p-JNK (1:1,000, 80024-1-RR; Proteintech).

### Co-immunoprecipitation assay

HEK-293T cells were divided into three groups and transfected with FLAG-OMD, HA-ITGB8, FLAG-OMD or HA-ITGB8 for 48 h. Cells were lysed in a buffer containing 1 mM phenylmethylsulfonyl fluoride, 1 mM dithiothreitol and a protease inhibitor cocktail. Cell lysates were immunoprecipitated overnight at 4 °C with anti-FLAG (20543-1-AP; Proteintech) or anti-HA (51064-2-AP; Proteintech) antibodies, followed by incubation with Protein A/G beads at 4 °C for 3 h. The complexes were washed five times with PBS containing protease inhibitor cocktail at 4 °C, separated via 10% sodium dodecyl sulfate–polyacrylamide gel electrophoresis and subjected to WB.

### Seahorse extracellular flux analysis

The oxygen consumption rate (OCR) and extracellular acidification rate (ECAR) were measured using a Seahorse XFe96 extracellular flux analyzer (Agilent Technologies). An Agilent Seahorse XF Cell Mito Stress Test Kit (103015-100) was used for the assay. In brief, BMMs were seeded in Seahorse 96-well cell culture microplates at a density of 3 × 10^4^ cells per well. Osteoclasts were induced for 3 days using 100 ng/ml GST-RANKL and 30 ng/ml M-CSF. On the day of the experiment, the medium was replaced with unbuffered XF-DMEM (pH 7.4) (103575-100; Agilent Technologies) containing 10 mM glucose, 200 ng/ml GST-RANKL, 100 ng/ml M-CSF, 1 mM pyruvate and 2 mM glutamine. Cells were equilibrated in a non-CO_2_ incubator at 37 °C for 1 h. For OCR assessment, sequential injections of oligomycin (1 μM), FCCP (2 μM), rotenone (1 μM) and antimycin A (1 μM) were performed according to the manufacturer’s protocol. ECAR was detected using a sequential injection of 10 mM glucose, 2 mM oligomycin and 50 mM 2-deoxyglucose. The ATP rate was determined using a sequential injection of oligomycin (1 μM), rotenone (1 μM) and antimycin A (1 μM).

### Glucose uptake assays

BMMs were serum-starved overnight and subsequently stimulated for 5 h in the presence or absence of OMD. After removal of OMD, cells were incubated in glucose-free medium for 30 min, followed by incubation with the fluorescent glucose analog 2-NBDG (100 μg/ml) at 37 °C for an additional 30 min. Cells were then washed twice with PBS, and nuclei were counterstained with Hoechst. Fluorescent images were acquired using a fluorescence microscope (Leica).

### Analysis of mitochondrial membrane potential

After 2 days of the indicated treatments, osteoclast precursors were washed three times with prewarmed α-MEM at 37 °C and then incubated with 500 μl of 1× TMRE working solution (C2001S, Beyotime) at 37 °C for 30 min in the dark. Cells were subsequently washed six times with prewarmed α-MEM. Fluorescent images were acquired using a fluorescence microscope (Leica).

### RNA sequencing and analysis

Total RNA was extracted using TRIzol reagent (15596018; Thermo Fisher Scientific) following the manufacturer’s protocol. RNA quantity and purity were assessed using a Bioanalyzer 2100 and RNA 6000 Nano LabChip Kit (5067-1511; Agilent). High-quality RNA samples with an RNA integrity number >7.0 were selected for sequencing library preparation. After RNA extraction, mRNA was purified from 5 µg of total RNA using Dynabeads Oligo (dT) (Thermo Fisher Scientific), followed by two rounds of purification. Paired-end sequencing (PE150) was performed using an Illumina NovaSeq 6000 (LC-Bio Technology) according to the manufacturer’s guidelines.

For read alignment, HISAT2 was used to map all samples’ reads to the mouse reference genome. The software first filtered out low-quality reads based on the associated quality scores and then aligned the remaining reads to the reference genome, allowing up to 20 multiple alignments per read (default settings) and permitting up to two mismatches during mapping. HISAT2 also builds a potential splice site database, confirming these mappings by comparing previously unmapped reads with the inferred junction database. The mapped reads for each sample were assembled using StringTie with default parameters.

Differential gene expression analysis was performed using DESeq2 software (edgeR for pairwise comparisons between two samples). Genes with a false discovery rate <0.05 and an absolute fold change ≥2 were considered differentially expressed. Enrichment analyses of Gene Ontology (GO) terms and Kyoto Encyclopedia of Genes and Genomes (KEGG) pathways were conducted for the differentially expressed genes. Gene Set Enrichment Analysis (v4.1.0) and MSigDB software were used to evaluate whether specific GO terms and KEGG pathways showed significant differences between the groups. In brief, the gene expression matrix was ranked using the Signal2Noise normalization method. Enrichment scores and *P* values were computed using default parameters. All bioinformatic analyses were performed using OmicStudio.

### LC–MS/MS analysis

Liquid chromatography (LC)–tandem mass spectrometry (MS/MS) experiments were performed using a Q Exactive Plus mass spectrometer coupled to an Easy nLC1200 (Thermo Scientific). Peptides were loaded onto a trap column (100 μm × 20 mm, 5 μm C18, Dr. Maisch GmbH) in buffer A (0.1% formic acid in water) and separated using reverse-phase high-performance LC with a self-packed column (75 μm × 150 mm; 3 μm ReproSil-Pur C18 beads, 120 Å, Dr. Maisch GmbH) at a flow rate of 300 nl/min. The reverse-phase high-performance LC gradient consisted of: 2%-4% buffer B (0.1% formic acid in 95% acetonitrile) from 0 to 2 min, 4%-30% buffer B from 2 to 47 min, 30%-45% buffer B from 47 to 52 min, 45%-90% buffer B from 52 to 54 min and 90% buffer B maintained until 60 min. MS data were acquired using a data-dependent top 20 method, dynamically selecting the most abundant precursor ions from the survey scan (350-1800 *m*/*z*) for high-energy collisional dissociation fragmentation. A lock mass of 445.120025 Da was used as the internal standard for mass calibration. Full MS scans were acquired at resolutions of 70,000 (*m*/*z* 200), with MS/MS scans at 15,000 resolution. Maximum injection times were set to 50 ms for MS and 25 ms for MS/MS. The normalized collision energy was 28, and the isolation window was set to 1.6 Th. The duration of the dynamic exclusion was 30 s.

### Nontargeted metabolomics analysis

Cell samples were quenched and metabolites were extracted using 1 ml of precooled methanol/acetonitrile/water (2:2:1, v/v/v). The mixture was sonicated for 1 h in an ice bath, incubated at −20 °C for 1 h and centrifuged at 14,000*g* for 20 min at 4 °C. The supernatants were collected, dried under vacuum and reconstituted in 50% acetonitrile before LC-MS analysis. Quality control (QC) samples were prepared by pooling equal aliquots of all samples and processed identically.

Metabolomic profiling was performed using an UHPLC system (Agilent 1290 Infinity LC) coupled to a TripleTOF 5600 mass spectrometer (AB Sciex) with electrospray ionization operated in both positive and negative modes. Metabolites were separated on an ACQUITY UPLC BEH Amide column (2.1 × 100 mm, 1.7 μm) with a flow rate of 0.5 ml/min. The mobile phase consisted of 25 mM ammonium acetate and 25 mM ammonium hydroxide in water (A) and acetonitrile (B), with a gradient elution. MS data were acquired over an *m*/*z* range of 60-1200 for MS and 25-1200 for MS/MS using information-dependent acquisition.

Raw data were converted to mzXML format using ProteoWizard and processed with XCMS for peak detection, retention time correction and alignment. Metabolites were annotated on the basis of accurate mass (<25 ppm) and MS/MS spectral matching. Multivariate analyses including principal component analysis and orthogonal partial least squares discriminant analysis were performed using SIMCA software with Pareto scaling. Differential metabolites were identified on the basis of variable importance on projection >1.0 and *P* < 0.05 (Student’s *t*-test or analysis of variance (ANOVA)). Differential features were identified using prespecified thresholds (|log_2_ fold change| and false discovery rate-adjusted *P* values).

### Homology modeling and molecular docking

A three-dimensional (3D) model of OMD was established using the SWISS-MODEL online tool with the template PDB ID 5YQ5. Sequence alignment revealed that the sequence similarity between the target protein OMD and the template was 80.15%. The Global Model Quality Estimation was 0.70 and the QMEAN score was 0.72, indicating that the structure was reliable and suitable for computational experiments. The 3D structure of ITGB8 predicted using AlphaFold was downloaded from the UniProt database. Protein-protein docking calculations were performed using the HDOCK online tool. The top ten models were output as the optimal conformations.

### OVX- and LPS-induced osteoporosis model

An OVX-induced osteoporosis model was established as described previously^26^. Twelve-week-old female C57BL/6 mice underwent sham or OVX surgery. Mice were randomly assigned to four groups: sham surgery, OVX saline injection, OVX osalmid injection and OVX OMD injection. Saline, osalmid (100 mg/kg via intraperitoneal injection) or OMD (200 μg/kg via intravenous injection) was administered three times per week for 6 weeks. Seven weeks after surgery, all mice were euthanized. Femurs and tibias were collected, fixed in 4% paraformaldehyde and subjected to micro-CT and bone histomorphometric analyses.

An LPS-induced osteolysis model, as previously described^31^, was established. Twelve-week-old female C57BL/6 mice were randomly allocated to four groups: sham surgery, LPS plus saline injection, LPS plus osalmid injection and LPS plus OMD injection. Saline, osalmid (100 mg/kg via intraperitoneal injection) or OMD (200 μg/kg via intravenous injection) were administered daily for 1 week. After the treatment period, all mice were euthanized. Femurs and tibias were collected, fixed in 4% paraformaldehyde and subjected to micro-CT and bone histomorphometric analysis.

### Bioinformatics analysis of public datasets

Transcriptome data were retrieved from the GEO database and analyzed. The datasets included gene expression profiles of mesenchymal stem cells in patients with primary osteoporosis and control group (GSE35958), gene expression profiles of pre-osteoblasts under simulated microgravity-induced inhibition of differentiation (GSE1367), gene expression analysis from bone biopsies of patients with endogenous Cushing’s syndrome before and after treatment (GSE30159) and gene expression profiling during RANKL-mediated osteoclast differentiation (GSE176265).

### Statistical analysis

Statistical analyses were performed using GraphPad Prism 8 software (GraphPad Software). All data are presented as mean ± standard error of the mean (s.e.m.), with *P* < 0.05 considered statistically significant. An unpaired *t*-test was used for comparisons between two experimental groups. One-way or two-way ANOVA was used to determine the statistical significance among multiple groups. Pearson’s correlation analysis was used to calculate correlation coefficients. All in vitro experiments were independently repeated at least three times. All in vitro replication attempts were successful for in vitro experiments. All in vivo experiments were performed using at least five biologically independent animals in each group. All replication attempts were successful in vivo.

## Results

### OMD decreases in osteoporosis and is negatively correlated with bone loss

**Fig. 1:**
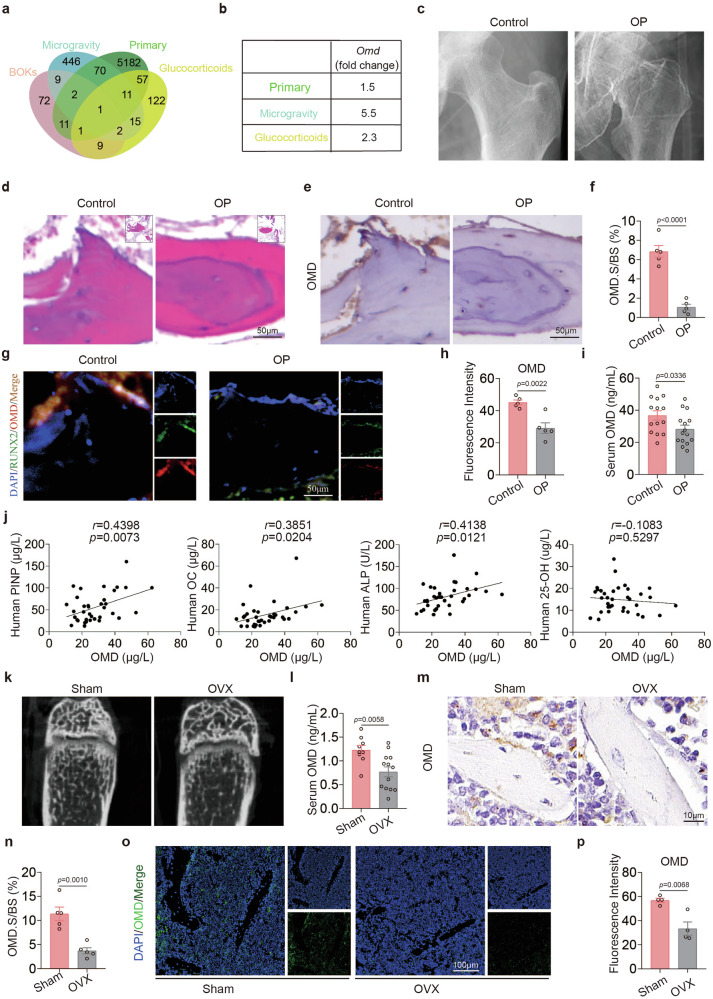
OMD is negatively correlated with bone loss. **a** Venn diagram illustrating the intersection of the downregulated differentially expressed genes in the osteoporosis group across three osteoporosis sequencing datasets (GSE30159, GSE35958 and GSE1367) and BOKs (fold change >1.5, *P* < 0.05). **b** Fold changes of *Omd* downregulation in the osteoporotic groups across the three sequencing datasets. **c** Representative X-ray images of femurs from controls and patients with osteoporosis. **d** H&E staining of the femurs from controls and patients with osteoporosis. Scale bar, 100 μm. **e**,**f** Representative images of OMD immunohistochemical staining in the femur (**e**) and quantification of the percentage of OMD-positive areas (**f**) (*n* = 5). Scale bar, 50 μm. **g**,**h** Immunofluorescence staining of OMD (red) and RUNX2 (green) (**g**) with quantitative analysis of the images (**h**) (*n* = 5). Scale bar, 50 μm. **i**, OMD levels in the serum of postmenopausal osteoporotic women (*n* = 15) and the control group (*n* = 14). **j** Correlation analysis of OMD levels in female serum with PINP, OC, ALP and 25-OH (*n* = 36). **k** Representative reconstructed 3D micro-CT images of the femurs of sham and OVX mice (*n* = 5). **l**, OMD levels in the serum of sham (*n* = 9) and OVX (*n* = 13) mice. **m**,**n** Representative images of OMD immunohistochemical staining in the distal femur (**m**) and quantification of the percentage of OMD-positive areas (**n**) (*n* = 5). Scale bar, 10 μm. **o**,**p** Immunofluorescence staining of OMD (**o**) and quantitative analysis of the images (**p**) (*n* = 4). Scale bar, 100 μm. Data represent mean ± s.e.m. Experimental data for each quantitative analysis were replicated at least five times. Statistical significance was assessed using unpaired *t*-tests (**f**, **h**, **i**, **l**, **n** and **p**) or Pearson’s correlation analysis (**j**).

The occurrence and progression of osteoporosis are closely related to an imbalance in bone metabolism, which is mainly mediated by BOKs^5^. Therefore, to further explore the key BOKs in bone metabolism, we analyzed sequencing data from three published osteoporosis models: glucocorticoid-induced osteoporosis (Cushing’s syndrome), primary osteoporosis and microgravity-induced bone loss (GSE30159, GSE35958 and GSE1367). Based on these datasets, we performed a joint analysis of genes significantly downregulated (fold change >1.5) in the osteoporosis groups and identified BOKs^5^ (Fig. 1a). The results revealed that the bone-specific osteogenic factor *Omd* was the only gene commonly and significantly downregulated across all osteoporosis groups (Fig. 1b). Therefore, we aimed to further investigate the relationship between OMD and osteoporosis.

We evaluated OMD levels in bone tissue from postmenopausal women with osteoporotic femoral neck fractures (undergoing hip replacement surgery) and compared these levels with age- and sex-matched postmenopausal women without osteoporosis (undergoing hip arthroplasty for hip osteoarthritis) (Fig. 1c,d). Immunohistochemical and immunofluorescence analyses revealed a significant decrease in OMD expression in osteoporotic bone tissue compared with controls (Fig. 1e-h). Furthermore, colocalization staining with RUNX2 confirmed the osteoblastic origin of OMD, aligning with findings from prior research^32^. We further measured serum OMD levels in postmenopausal women with osteoporosis and compared them with those in age-matched control participants without osteoporosis. The findings indicated a 23.6% reduction in serum OMD concentration in the postmenopausal osteoporotic group compared with the controls (36.78 ± 10.91 ng/ml versus 28.17 ± 9.80 ng/ml) (Fig. 1i). It has been shown that postmenopausal osteoporosis is marked by excessive osteoclast formation, leading to bone loss^33^. We hypothesized that decreased OMD might be responsible for increased osteoclast formation. Moreover, we evaluated OMD levels and bone metabolic markers in the serum of women across different age groups. The results showed a significant positive correlation between OMD and procollagen type I N-terminal propeptide (PINP), osteocalcin (OC) and alkaline phosphatase (ALP) (Fig. 1j). However, no association was observed between OMD and Ca^2+^ or 25-hydroxyvitamin D (25-OH) (Fig. 1j and Supplementary Fig. 1a).

To confirm the relationship between OMD and bone metabolism, an OVX-induced osteoporosis mouse model was used (Fig. 1k). Serum OMD levels in the OVX mice were significantly lower than those in the sham group (Fig. 1l). Immunohistochemistry and immunofluorescence results from the distal femur of mice demonstrated reduced OMD levels in the OVX group (Fig. 1m-p). The ELISA assay results indicate that the OMD levels in mouse serum were negatively correlated with β-CTX (Supplementary Fig. 1b), with β-CTX being a key indicator of osteoclastic activity^34^. Collectively, these findings suggest a negative correlation between OMD and postmenopausal bone loss, indicating a potential role for OMD in the pathogenesis of osteoporosis.

### OMD deficiency leads to significant bone loss and enhanced osteoclast formation in vivo

**Fig. 2:**
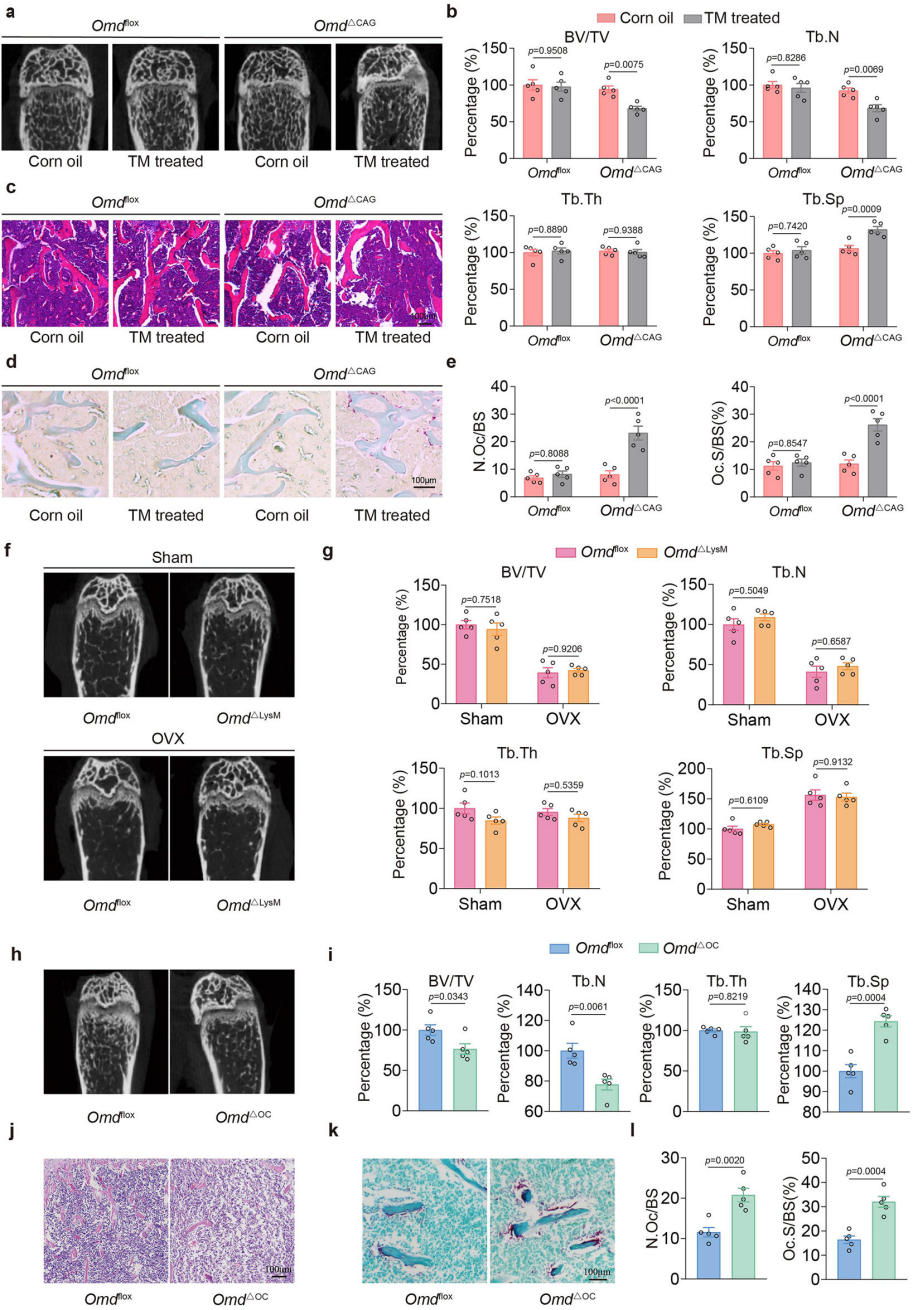
OMD deficiency leads to significant bone loss and enhanced osteoclast formation in vivo. **a** Representative reconstructed 3D micro-CT images of the femurs in *Omd*^flox^ and *Omd*^Δ CAG^ mice treated with corn oil (vehicle) or tamoxifen (*n* = 5). **b** Quantification of BV/TV, Tb.N, Tb.Th and Tb.Sp from micro-CT images (*n* = 5). **c** H&E staining of the femurs from 3-month-old *Omd*^flox^ and *Omd*^Δ CAG^ mice treated with corn oil (vehicle) or tamoxifen (*n* = 5). Scale bar, 100 μm. **d** TRAP staining of the femurs from 3-month-old *Omd*^flox^ and *Omd*^Δ CAG^ mice treated with corn oil (vehicle) or tamoxifen (*n* = 5). Scale bar, 100 μm. **e** Quantification of N.Oc/BS and Oc.S/BS (%) (*n* = 5). **f** Representative reconstructed 3D micro-CT images of the femurs of 3-month-old *Omd*^flox^ and *Omd*^ΔLysM^ mice (*n* = 5). **g** Quantification of BV/TV, Tb.N, Tb.Th and Tb.Sp from micro-CT images (*n* = 5). **h** Representative reconstructed 3D micro-CT images of the femurs of 3-month-old *Omd*^flox^ and *Omd*^ΔOC^ mice (*n* = 5). **i** Quantification of BV/TV, Tb.N, Tb.Th and Tb.Sp from micro-CT images (*n* = 5). **j** H&E staining of the femurs from 3-month-old *Omd*^flox^ and *Omd*^ΔOC^ mice (*n* = 5). Scale bar, 100 μm. **k** TRAP staining of the femurs from 3-month-old *Omd*^flox^ and *Omd*^ΔOC^ mice (*n* = 5). Scale bar, 100 μm. **l** Quantification of N.Oc/BS and Oc.S/BS (%) (*n* = 5). Data represent mean ± s.e.m. Experimental data for each quantitative analysis were replicated at least five times. Statistical significance was assessed using unpaired *t*-tests (**i** and **l**) or two-way ANOVA (**b**, **e** and **g**).

To investigate the role of OMD in bone metabolism, we generated tamoxifen-induced OMD gKO mice (*Omd*^ΔCAG^) (Supplementary Fig. 2a-c), and femurs were collected from 3-month-old male mice to assess bone mass. First, because tamoxifen can influence bone remodeling^35^, age- and sex-matched *Omd*^flox^ littermates were subjected to the identical tamoxifen/vehicle regimen and analyzed in parallel. The results showed that, under our experimental conditions, short-term tamoxifen exposure in *Omd*^flox^ controls did not significantly alter trabecular parameters or osteoclast indices (Fig. 2a-e and Supplementary Fig. 2a,b). Then, compared with the corn oil control group, quantitative micro-CT analysis demonstrated a significant reduction in trabecular bone mass in *Omd*^ΔCAG^ mice injected with tamoxifen (Fig. 2a), as indicated by decreased BV/TV and Tb.N, and increased Tb.Sp associated with osteoclastic activity (Fig. 2b). H&E staining also revealed a significantly decreased trabecular number in the distal femur of *Omd*^ΔCAG^ mice injected with tamoxifen (Fig. 2c). Furthermore, TRAP staining confirmed a higher number of osteoclasts along the trabecular surface in *Omd*^ΔCAG^ mice injected with tamoxifen (Fig. 2d,e). These findings indicate that global OMD deficiency enhances osteoclast formation, resulting in bone loss.

To determine whether osteoclast formation was impacted by endogenous OMD, we subsequently generated *Omd*^ΔLysM^ conditional knockout mice and confirmed efficient *Omd* deletion by tail genotyping and immunofluorescence staining of isolated BMMs (Supplementary Fig. 2d,e). *Omd*^*Δ*LysM^ mice underwent OVX at 8 weeks of age, followed by observation for 14 weeks after recovery. Micro-CT analysis indicated that OMD deletion in osteoclast precursors did not lead to significant alterations in the trabecular bone mass (Fig. 2f). There were no notable differences in the BV/TV, Tb.N, Tb.Sp or Tb.Th (Fig. 2g). H&E staining results showed no significant trabecular loss in the distal femoral trabeculae of *Omd*^*Δ*LysM^ mice compared with those of *Omd*^flox^ mice (Supplementary Fig. 3a). Similarly, no notable differences were observed in TRAP staining between the two groups (Supplementary Fig. 3b,c). We further examined the relationship between OMD levels in osteoclast precursor cells and osteoclast differentiation. The results demonstrated that OMD levels in osteoclast precursors did not change significantly during osteoclast differentiation (Supplementary Fig. 3d,e), aligning with previous sequencing results (GSE176265) (Supplementary Fig. 3f). These results might have suggested that the osteoclast differentiation process had a low dependency on OMD in osteoclast precursor cells and indicated that the specific knockout of OMD in osteoclast precursors did not significantly affect osteoclast formation or bone mass.

OMD is predominantly secreted by mature osteoblasts^32^. We analyzed the published single-cell sequencing data of primary cells from the mouse calvaria^36^ and found that OMD is mainly expressed in osteoblasts and chondrocytes (Supplementary Fig. 3g). Therefore, osteoblast-specific OMD knockout (*Omd*^*Δ*OC^ and *Omd*^*Δ*DMP1^) mice were used to investigate the role of OMD in regulating bone mass and osteoclastic activity (Supplementary Fig. 2f-i). Interestingly, consistent with obervation in the gKO mice, micro-CT analysis demonstrated that both *Omd*^*Δ*OC^ and *Omd*^*Δ*DMP1^ mice exhibited a significant reduction in trabecular bone mass (Fig. 2h; Supplementary Fig. 4a), BV/TV and Tb.N, along with a marked increase in Tb.Sp compared with *Omd*^flox^ mice (Fig. 2i and Supplementary Fig. 4b). H&E and TRAP staining of the distal femoral trabeculae in OMD deletion mice revealed reduced cancellous bone tissue and an increased number of osteoclasts (Fig. 2j-l and Supplementary Fig. 4c-e). These findings suggest that specific deletion of OMD in osteoblasts promotes osteoclastic activity and subsequent bone loss.

### Both endogenous and exogenous OMD can regulate osteoclast differentiation in vitro

**Fig. 3:**
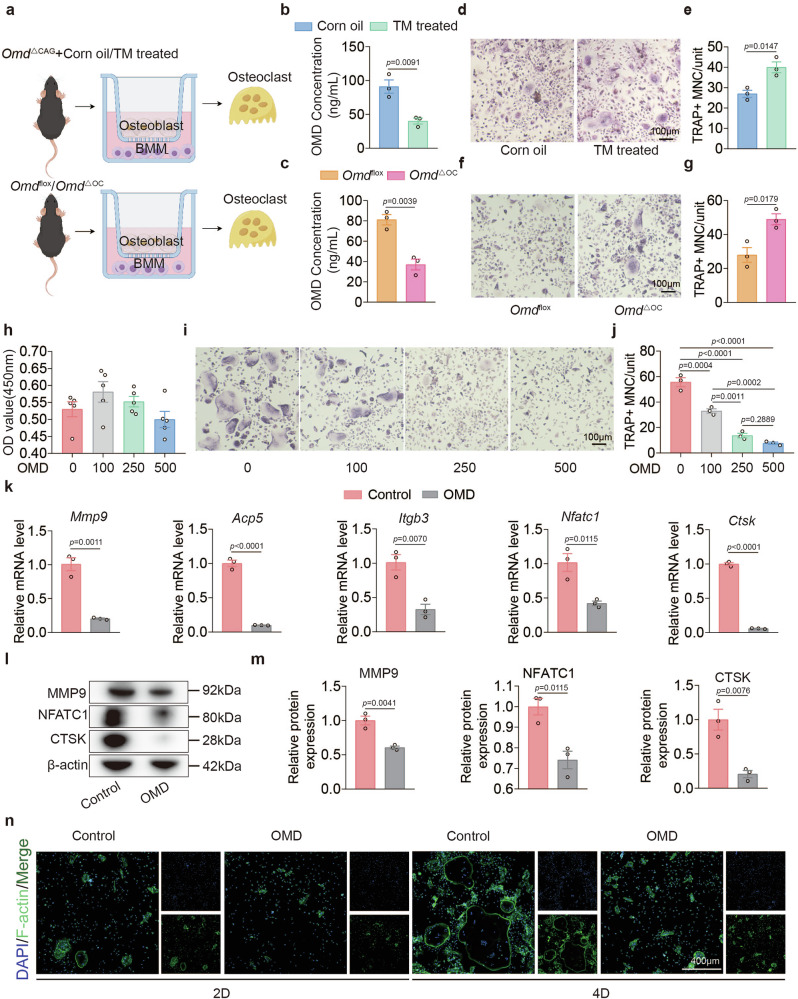
Both endogenous and exogenous OMD can regulate osteoclast differentiation in vitro. **a** Osteoblasts extracted from the femurs of *Omd*^ΔCAG^ mice injected with corn oil, *Omd*^ΔCAG^ mice injected with tamoxifen, *Omd*^flox^ mice or *Omd*^ΔOC^ mice were cocultured with BMMs from wild-type mice to induce osteoclastogenesis. **b**,**c** The supernatant from the coculture medium was collected to assess the concentration of OMD in *Omd*^ΔCAG^ (**b**), *Omd*^Δflox^ and *Omd*^ΔOC^ (**c**) group (*n* = 3). **d**–**g** TRAP staining was performed on osteoclasts from group *Omd*^ΔCAG^ (**d**), *Omd*^Δflox^ and *Omd*^ΔOC^ (**f**) after 9 days of coculture, followed by a quantitative analysis of the nucleus counts in TRAP-positive multinuclear cells in group *Omd*^ΔCAG^ (**e**), *Omd*^Δflox^ and *Omd*^ΔOC^ (**g**) (*n* = 3). Scale bar, 100 μm. **h** Effects of recombinant OMD on BMMs viability at 48 h (*n* = 5). **i**,**j** After a 4-day treatment with 30 ng/ml M-CSF and 100 ng/ml RANKL, TRAP staining was conducted on BMMs exposed to various concentrations of recombinant OMD (**i**), followed by a quantitative analysis of the nuclei counts in TRAP-positive multinuclear cells (**j**) (*n* = 3). Scale bar, 100 μm. **k** After a 1-day induction, the mRNA levels of osteoclastogenic genes were evaluated by qRT–PCR in BMMs treated with 100 ng/ml recombinant OMD protein and in the control group (*n* = 3). **l**,**m** After a 3-day induction, the protein levels of osteoclastogenic genes were assessed by WB in BMMs treated with 100 ng/ml recombinant OMD protein and in the control group (**l**), followed by quantitative analysis of the proteins (**m**) (*n* = 3). **n** After a 2-day or 4-day induction, the F-actin rings in the control group and in BMMs treated with 100 ng/ml recombinant OMD protein were observed using phalloidin staining (*n* = 3). Scale bar, 400 μm. Data represent mean ± s.e.m. Experimental data for each quantitative analysis were replicated at least three times. Statistical significance was assessed using unpaired *t*-tests (**b**, **c**, **e**, **g**, **k** and **m**) or one-way ANOVA (**h** and **j**).

To model osteoblast-to-osteoclast precursor paracrine signaling, we isolated osteoblasts from tamoxifen-treated *Omd*^ΔCAG^ and *Omd*^ΔOC^ mice and their littermate controls, cultured them in the upper chamber of a transwell chamber and measured the concentration of OMD in the culture supernatant 1 day later (Fig. 3a). The results indicated that the concentration of OMD secreted by osteoblasts from tamoxifen-injected *Omd*^ΔCAG^ and *Omd*^ΔOC^ mice was significantly lower than that from corn-oil-injected *Omd*^ΔCAG^ mice or *Omd*^flox^ mice (Fig. 3b,c). Subsequently, BMMs from wild-type mice were cocultured with osteoblasts in the lower chamber of a transwell system to induce osteoclastogenesis. After 9 days of coculture in a medium containing 10 nM vitamin D3, TRAP staining was performed to assess osteoclast formation. The results demonstrated that BMMs cocultured with osteoblasts from tamoxifen-treated or *Omd*^ΔOC^ mice exhibited an enhanced osteoclast differentiation capacity (Fig. 3d-g). Similarly, after 3 days of coculture, the transcription levels of osteoclast differentiation markers were higher in the knockout group than in the control group (Supplementary Fig. 5a,b). These findings suggest that reduced OMD secretion by osteoblasts promotes osteoclast differentiation.

To further verify the role of OMD in osteoclastogenesis, we added recombinant OMD during the differentiation of BMMs into osteoclasts. The CCK-8 results indicated that the recombinant OMD protein at a concentration of 100 ng/ml exhibited no significant toxicity to BMMs, a concentration determined on the basis of observations from the aforementioned coculture experiment (Fig. 3b,c,h). TRAP staining demonstrated that OMD inhibited osteoclast formation in a concentration- and time-dependent manner (Fig. 3i,j and Supplementary Fig. 5c,d). We then treated BMMs with recombinant OMD protein at a concentration of 100 ng/ml. After 1 or 3 days of treatment with M-CSF and RANKL, we examined the transcriptional and protein levels of osteoclast differentiation markers. OMD-treated BMMs exhibited marked inhibition of osteoclast differentiation, with the most significant effect observed on the secretion of the key functional protein, CTSK, by osteoclasts (Fig. 3k-m). On days 2 or 4 of osteoclast differentiation induction, fluorescence staining of F-actin revealed marked suppression of filopodia formation in the OMD-treated group (Fig. 3n and Supplementary Fig. 5e). To further investigate whether OMD can inhibit LPS-induced osteoclastogenesis, we treated cells with various concentrations of OMD while simultaneously inducing osteoclast differentiation with 100 ng/ml LPS. After 24 h, we assessed the mRNA levels of osteoclast differentiation markers. Our results demonstrated that OMD significantly inhibited LPS-stimulated osteoclastogenesis in a concentration-dependent manner (Supplementary Fig. 5f). These data suggest that OMD inhibits osteoclast differentiation in vitro.

### OMD reduces mitochondrial respiration and mitochondrial ATP production during osteoclastogenesis

**Fig. 4:**
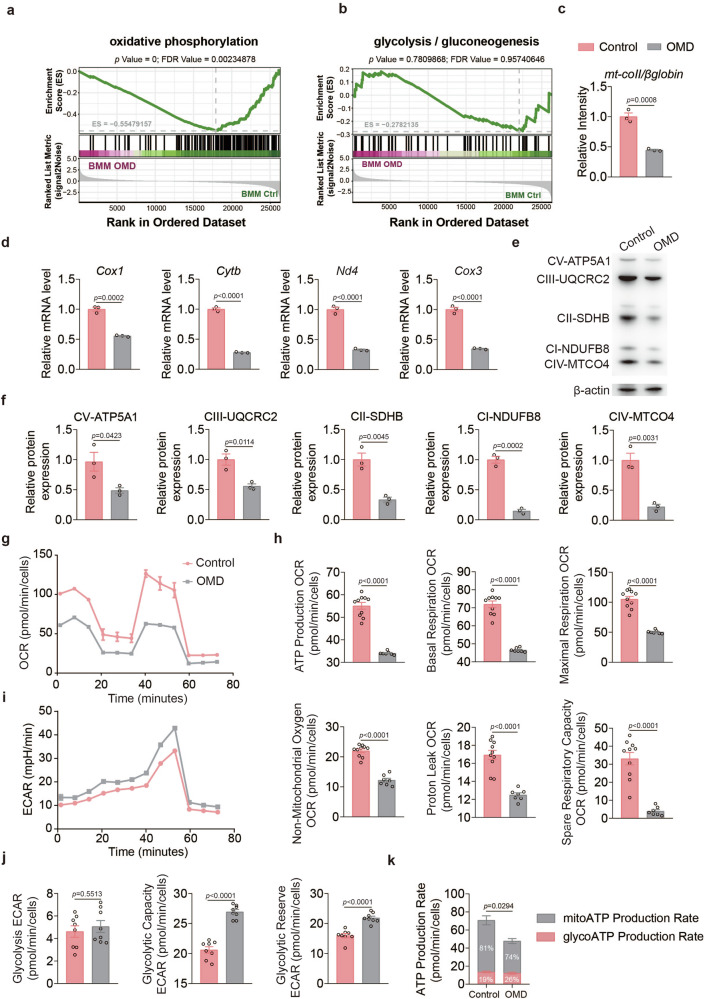
OMD reduces mitochondrial respiration and mitochondrial ATP in osteoclast differentiation. **a** The GSEA plot reveals the suppression of the OXPHOS signaling pathway in the OMD-treated group. **b** The GSEA plot reveals no significant differences in the glycolysis pathway between the control group and the OMD-treated group. **c** Relative abundance of mitochondria determined by quantitative PCR (qPCR) of *mt-Co2II* DNA normalized to β-globin (*n* = 3). **d** After a 1-day induction, the levels of mRNA associated with mitochondrial OXPHOS markers were measured in the treatment group with 100 ng/ml recombinant OMD protein and the control group using qRT–PCR (*n* = 3). **e**,**f** After a 3-day induction, the protein levels of mitochondrial OXPHOS complexes were assessed by WB in BMMs treated with 100 ng/ml recombinant OMD protein and in the control group (**e**), followed by quantitative analysis of the proteins (**f**) (*n* = 3). **g**,**h** After a 3-day induction, OCR was measured (**g**), including detailed parameters such as basal respiration, maximal respiration, ATP-linked respiration, spare respiratory capacity, nonmitochondrial respiration and proton leak (**h**). **i**,**j** After a 3-day induction, ECAR was measured (**i**), including detailed parameters such as glycolysis, glycolytic capacity and glycolytic reserve (**j**). **k** After a 3-day induction, the ATP production rate was measured (*n* = 3). Data represent mean ± s.e.m. Experimental data for each quantitative analysis were replicated at least three times. Statistical significance was assessed using unpaired *t*-tests (**c**, **d**, **f**, **h**, **j** and **k**).

To explore the mechanisms underlying OMD-mediated regulation of osteoclastogenesis, we conducted an unbiased whole-genome analysis to characterize the transcriptional changes in BMMs treated with or without OMD for 1 day. Gene set enrichment analysis (GSEA) of the RNA sequencing data indicated significant enrichment of oxidative phosphorylation (OXPHOS)-related genes between groups (Fig. 4a and Supplementary Fig. 6a). Meanwhile, glycolysis, another critical energy-generating pathway for osteoclast differentiation, exhibited no significant differences between the two groups (Fig. 4b). Given the significant alterations in CTSK observed following OMD treatment (Fig. 3k-m), which are intricately associated with mitochondrial respiration^37^, we further examined the effect of OMD on mitochondrial respiration.

Mitochondrial DNA (mtDNA) encodes the core components of the OXPHOS complex, making its stable expression essential for maintaining cellular energy homeostasis^38^. Thus, we first assessed the mitochondrial copy number to evaluate metabolic changes. The results indicated that OMD significantly suppressed the mtDNA copy number during osteoclast differentiation (Fig. 4c). Meanwhile, mitochondrial function-related genes regulated by mtDNA were also notably downregulated in the OMD-treated group, with *Cox1* decreasing by 44.5 ± 3.3%, *Cox3* by 65.2 ± 3.7%, *Cytb* by 72.1 ± 2.2% and *Nd4* by 67 ± 4.0% (Fig. 4d). In addition, compared with the control group, the OMD-treated group exhibited a significant reduction in the expression of proteins involved in the assembly and function of the electron transport chain (ETC), including ATP5A1, UQCRC2, SDHB, NDUFB8 and MTCO4 (Fig. 4e,f). In vivo immunofluorescence indicated that specific knockout of the *Omd* gene in osteoblasts led to an increase in NDUFB8 (complex I subunit) abundance in TRAP-positive osteoclasts and aligning with our in vitro bioenergetic measurements (Supplementary Fig. 6b). To further determine whether the reduced expression of these proteins alters mitochondrial energy metabolism, we measured the OCR as a surrogate for mitochondrial respiration. Notably, compared with the control group, OMD-treated BMMs exhibited significantly reduced basal respiration, ATP-linked respiration and spare respiration (Fig. 4g,h). In addition, ECAR analysis showed that OMD treatment did not significantly alter basal glycolysis, whereas both glycolytic capacity and glycolytic reserve exhibited modest increases (Fig. 4i,j). Further assessment of ATP production rates revealed a marked reduction in mitochondrial ATP generation in the OMD group, while glycolytic ATP production remained largely unchanged. Accordingly, the proportion of glycolytic ATP relative to total ATP was increased following OMD treatment (Fig. 4k). Consistently, glucose uptake did not differ between the two groups (Supplementary Fig. 6c), whereas OMD markedly decreased mitochondrial membrane potential (Supplementary Fig. 6d). To broaden the assessment of global metabolic remodeling, we performed untargeted metabolomics during osteoclastogenesis (*n* = 5 per group). OMD treatment was associated with coordinated metabolic changes indicative of mitochondrial and energetic stress, including robust accumulation of multiple acylcarnitines, together with a trend toward increased adenosine monophosphate (AMP) and oxidized glutathione (GSSG) (Supplementary Fig. 6e,f). These results indicate that OMD limits mitochondrial respiration and mitochondrial ATP production during osteoclastogenesis of osteoclast precursors.

### RRM2-mediated inhibition of mitochondrial respiration and mitochondrial ATP induced by OMD

**Fig. 5:**
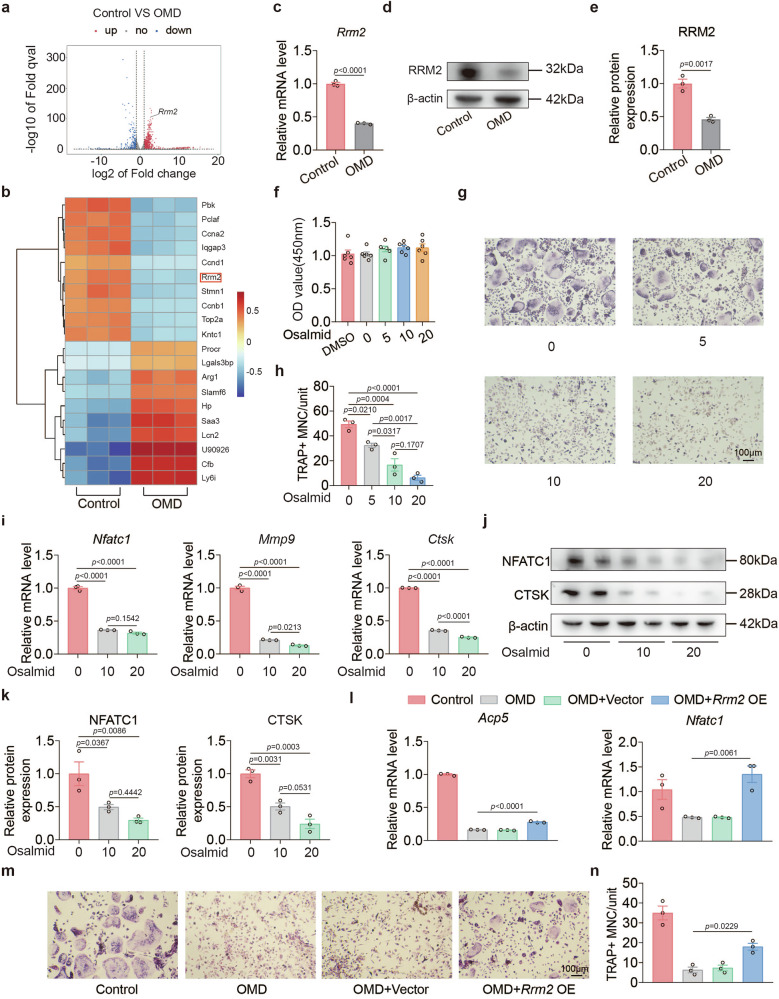
RRM2 is involved in the inhibition of osteoclastogenesis induced by OMD. **a** Volcano plot of all transcripts. Red dots indicate >2-fold change upregulation (*P* < 0.05) among the control group, while blue dots indicate >2-fold change downregulation (*P* < 0.05) among the control group. **b** The heat map illustrates the 20 most significantly differentially expressed genes between the control group and OMD-treated group. Red indicates upregulation, while blue indicates downregulation (*n* = 3). **c** After a 1-day induction, the mRNA levels of *Rrm2* were measured in both the treatment group (100 ng/ml recombinant OMD protein) and the control group using qRT–PCR (*n* = 3). **d**,**e** After a 3-day induction, the protein levels of RRM2 were assessed by WB in BMMs treated with 100 ng/ml recombinant OMD protein and in the control group (**d**), followed by quantitative analysis of the protein (**e**) (*n* = 3). **f** Effects of osalmid on BMMs viability at 48 h (*n* = 6). **g**,**h** After a 4-day induction, TRAP staining was conducted on BMMs exposed to various concentrations of osalmid (**g**) followed by a quantitative analysis of the nuclei counts in TRAP-positive multinuclear cells (**h**) (*n* = 3). Scale bar, 100 μm. **i** After a 1-day induction, the mRNA levels of osteogenic genes in BMMs treated with different concentrations of osalmid were assessed using qRT–PCR (*n* = 3). **j**,**k** After a 3-day induction, the protein levels of osteogenic genes in BMMs treated with varying concentrations of osalmid were analyzed using WB (**j**), followed by quantitative analysis of the proteins (**k**) (*n* = 3). **l** After treating BMMs with either a control vector or *Rrm2*-overexpressing adenovirus, different treatments were administered, and the mRNA levels of osteogenic genes were quantified using qRT–PCR (*n* = 3). **m**,**n** After a 4-day induction, TRAP staining was conducted on BMMs exposed to different treatments, followed by a quantitative analysis of the nuclei counts in TRAP-positive multinuclear cells (*n* = 3). Scale bar, 100 μm. Data represent mean ± s.e.m. Experimental data for each quantitative analysis were replicated at least three times. Statistical significance was assessed using unpaired *t*-test (**c** and **e**) or one-way ANOVA (**f**, **h**, **i**, **k**, **l** and **n**).

Having established that OMD reduces mitochondrial respiration and mitochondrial ATP production during osteoclastogenesis, we next investigated the downstream mechanisms underlying this bioenergetic phenotype. RNA sequencing identified *Rrm2* as one of the top 20 most differentially expressed genes (Fig. 5a,b). Consistent with the sequencing results, the expression level of *Rrm2* in the OMD-treated group decreased by nearly 60% compared with the control group (Fig. 5c-e). Because RRM2 contributes to deoxyribonucleotide synthesis required for mtDNA replication and maintenance, altered in *Rrm2* expression could plausibly influence mitochondrial function^39^. In parallel, metabolomic profiling revealed significant alterations in nucleotide-related metabolites following OMD treatment, particularly in deoxynucleotide-associated species. Notably, deoxycytidine and deoxyguanosine monophosphate were significantly dysregulated, suggesting impaired deoxynucleotide homeostasis, a process critically dependent on RRM2 activity (Supplementary Fig. 7a,b). Based on these convergent datasets, we postulated that *Rrm2* may serve as an important downstream node in the pathway by which OMD influences mitochondrial ATP production, and we therefore focused on testing the functional role of *Rrm2* in subsequent experiments.

To verify the role of RRM2 in osteoclastogenesis, we treated BMMs with the RRM2 inhibitor osalmid. The CCK-8 results indicated that osalmid at concentrations of 0-20 μM exhibited no significant toxicity toward BMMs (Fig. 5f). Subsequently, TRAP staining revealed that the same concentration range of osalmid inhibited osteoclast formation in a dose-dependent manner (Fig. 5g,h). Consistently, both mRNA and protein levels of osteoclast differentiation markers were significantly reduced compared with the saline-treated group (Fig. 5i-k). Notably, similar to the results observed after OMD treatment, the expression of CTSK was significantly inhibited following the suppression of RRM2 (Fig. 5i-k). To demonstrate the role of RRM2 in the OMD-mediated inhibition of osteoclastogenesis, we overexpressed *Rrm2* in BMMs following adenoviral treatment. The transcriptional levels of osteoclastic differentiation markers in the *Rrm2* overexpression group were significantly higher than those in the OMD treatment group (Fig. 5l). Meanwhile, overexpression of *Rrm2* partially rescued the decline in osteoclast formation induced by OMD, as indicated by the increase number of TRAP-positive cells in the in vitro osteoclastogenesis assays (Fig. 5m,n). In femoral sections from *Omd*^ΔOC^ mice, the RRM2 fluorescence intensity in TRAP-positive cells was significantly weaker compared with controls, further validating the role of RRM2 (Supplementary Fig. 7c).

**Fig. 6:**
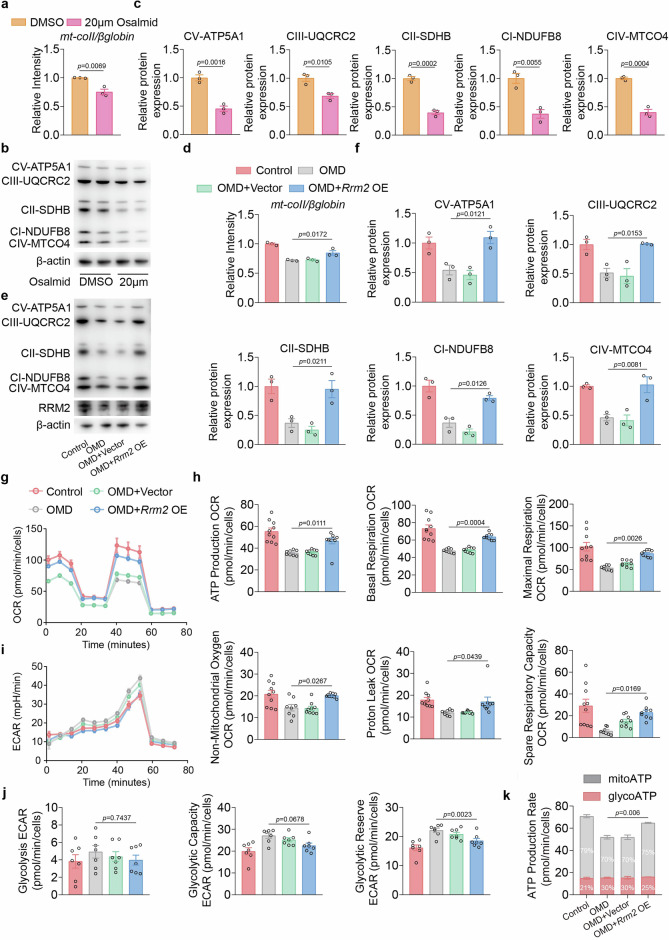
RRM2 is involved in the regulation of mitochondrial respiration and mitochondrial ATP during the OMD-mediated inhibition of osteoclast differentiation. **a** Relative abundance of mitochondria determined by qPCR of mt-Co2Ⅱ DNA normalized to β-globin (*n* = 3). **b**,**c** After a 3-day induction, the protein levels of mitochondrial OXPHOS complexes were assessed by WB in BMMs treated with 20 μm osalmid and DMSO (**b**), followed by quantitative analysis of the proteins (**c**) (*n* = 3). **d** Relative abundance of mitochondria determined by qPCR of mt-Co2Ⅱ DNA normalized to β-globin (*n* = 3). **e**,**f** After treating BMMs with either a control vector or *Rrm2*-overexpressing adenovirus, different treatments were applied, and 3 days post-osteoclastogenesis induction, the protein levels of mitochondrial OXPHOS complexes were assessed by WB (**e**), followed by quantitative analysis of the protein (**f**) (*n* = 3). **g**,**h** After a 3-day induction, OCR was measured (**g**), including detailed parameters such as basal respiration, maximal respiration, ATP-linked respiration, spare respiratory capacity, nonmitochondrial respiration and proton leak (**h**). **i**,**j** After a 3-day induction, ECAR was measured (**i**), including detailed parameters such as glycolysis, glycolytic capacity and glycolytic reserve (**j**). **k** After a 3-day induction, the ATP production rate was measured (*n* = 3). Data represent mean ± s.e.m. Experimental data for each quantitative analysis were replicated at least three times. Statistical significance was assessed using unpaired *t*-tests (**a** and **c**) or one-way ANOVA (**d**, **f**, **h**, **j** and **k**).

To further validate that RRM2 is responsible for OMD-mediated osteoclastogenesis through the inhibition of mitochondrial ATP, we showed that 20 μM of osalmid can downregulate mtDNA copy number (Fig. 6a). Simultaneously, BMMs treated with this dose showed a reduced expression of key proteins related to the assembly and function of the ETC, including ATP5A1, UQCRC2, SDHB, NDUFB8 and MTCO4 (Fig. 6b,c). By contrast, BMMs overexpressing *Rrm2* showed a restoration of mtDNA copy number after OMD treatment compared to BMMs treated with OMD alone (Fig. 6d). Consistently, key proteins involved in ETC composition and function were restored in the *Rrm2* overexpression group, including ATP5A1, UQCRC2, SDHB, NDUFB8 and MTCO4 (Fig. 6e,f). To examine whether specific ETC complexes affected osteoclast formation, we measured the OCR in different cell groups. *Rrm2* overexpression partially rescued the OMD-induced decline in mitochondrial respiratory capacity, with basal and ATP-linked respiration and restored spare respiratory capacity (Fig. 6g,h). Moreover, ECAR analysis showed that *Rrm2* overexpression in OMD-treated cells did not affect basal glycolysis or glycolytic capacity, but resulted in a modest reduction in glycolytic reserve (Fig. 6I,j). Compared with the OMD group, cells overexpressing *Rrm2* exhibited an overall increase in ATP production rates, which was primarily attributable to the restoration of mitochondrial ATP generation, whereas glycolytic ATP production showed minimal changes (Fig. 6k). Glucose uptake assays further demonstrated that *Rrm2* reconstitution did not alter glucose uptake rates (Supplementary Fig. 7d). Notably, the OMD-induced decrease in mitochondrial membrane potential was partially rescued by *Rrm2* overexpression (Supplementary Fig. 7e). Collectively, these results indicate that RRM2 mediates the inhibition of osteoclastogenesis by OMD through the suppression of mitochondrial respiration and mitochondrial ATP.

### The OMD-ITGB8-RhoA-YAP/TEAD axis represses *Rrm2* transcription and slows osteoclast differentiation

**Fig. 7:**
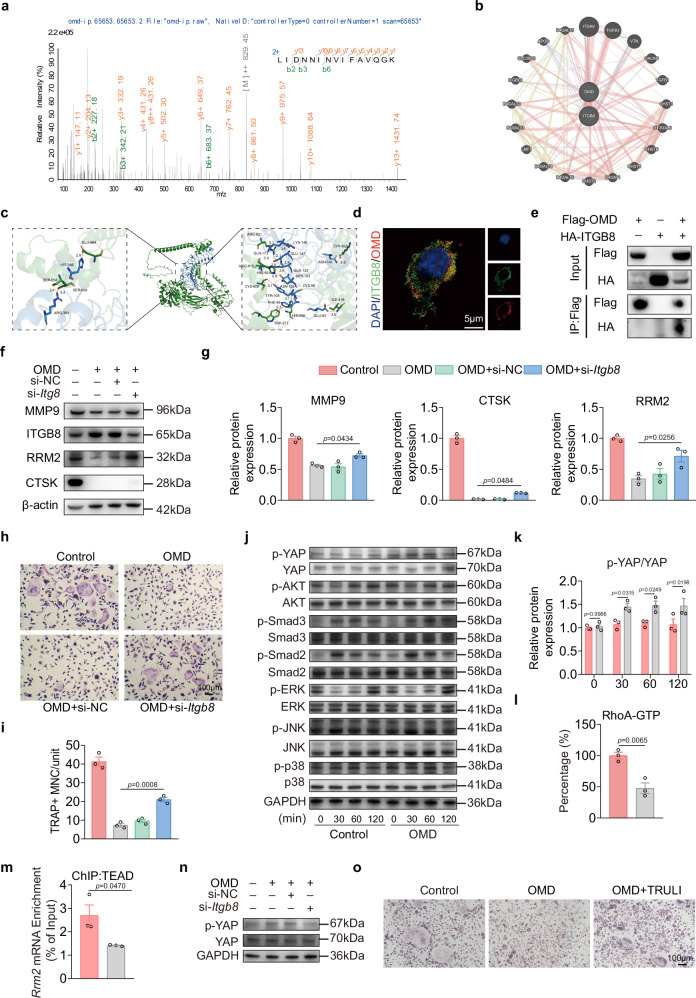
The OMD-ITGB8-RhoA-YAP/TEAD axis represses *Rrm2* transcription and slows osteoclast differentiation. **a** The MS of integrin β8 (ITGB8). **b** Predicted interactions between OMD and ITGB8 based on the GeneMANIA database. **c** The detailed interaction network between OMD and ITGB8. The key residues of OMD (in blue) and ITGB8 (in green) are displayed as sticks, with residue chain identifiers indicated. Dashed yellow lines represent hydrogen bonds, and dashed red lines indicate π–π interactions, with distances labeled. **d** Representative immunofluorescence images showing colocalization of OMD/ITGB8 in BMMs treated with 100 ng/ml OMD. Scale bar, 5 μm. **e** HEK-293T cells were transfected with Flag-OMD, HA-ITGB8 or Flag-OMD and HA-ITGB8. Flag immunoprecipitates were analyzed by immunoblotting as outlined. **f**,**g** After treating BMMs with either control siRNA or si-*Itgb8*, different treatments were applied, and 3 days after osteoclastogenesis induction, the protein levels of osteoclastogenesis markers, RRM2 and ITGB8 were assessed by WB (**f**), followed by quantitative analysis of the proteins (**g**) (*n* = 3). **h**,**i** After a 4-day induction, TRAP staining was conducted on BMMs exposed to different treatments (**h**), followed by a quantitative analysis of the nuclei counts in TRAP-positive multinuclear cells (**i**) (*n* = 3). Scale bar, 100 μm. **j**,**k** The levels of total and phosphorylated proteins in multiple signaling pathways at indicated time points (0, 30, 60 and 120 min) following OMD treatment were assessed by WB (**j**), followed by quantitative analysis of the proteins (**k**) (*n* = 3). **l** RhoA-GTP levels were measured by ELISA following OMD treatment (*n* = 3). **m** The occupancy of TEAD at the *Rrm2* promoter was assessed by ChIP (*n* = 3). **n** BMMs were transfected with control siRNA or si-*Itgb8* and subsequently subjected to the indicated treatments; total and phosphorylated YAP levels were analyzed by WB (*n* = 3). **o** After a 4-day induction, TRAP staining was conducted on BMMs exposed to different treatments (*n* = 3). Scale bar, 100 μm. Data represent mean ± s.e.m. Experimental data for each quantitative analysis were replicated at least three times. Statistical significance was assessed using unpaired *t*-tests (**k**–**m**) and one-way ANOVA (**g** and **i**).

OMD, a protein secreted by osteoblasts, is involved in interactions between osteoblasts and osteoclasts. To elucidate how extracellular OMD signals to osteoclast precursors, we screened candidate surface receptors. After transfecting the FLAG-tagged OMD plasmid into RAW 264.7 cells, immunoprecipitation MS was used to identify proteins interacting with OMD. Our results revealed that the membrane receptor ITGB8 is a potential interacting partner of OMD (Fig. 7a). GeneMANIA also predicted this interaction (Fig. 7b). The molecular docking results also indicated a high likelihood of interactions between OMD and ITGB8 at their natural binding sites, primarily through hydrogen bonding (Fig. 7c). Subsequent immunofluorescence results further revealed the colocalization of OMD protein and the ITGB8 receptor on the surface of osteoclast precursor cells (Fig. 7d). The immunoprecipitation results also validated these interactions (Fig. 7e). Overall, these results suggest that ITGB8 may serve as a potential receptor through which OMD modulates signaling in osteoclast precursor cells.

Next, we assessed the role of ITGB8 in OMD-induced inhibition of osteoclastogenesis. siRNA was used to downregulate *Itgb8* expression in osteoclast precursor cells. WB results demonstrated that knocking down *Itgb8* in OMD-treated osteoclast precursor cells partially restored osteoclastogenesis markers, including MMP9 and CTSK, while significantly upregulating RRM2 expression. These data suggest that RRM2 functions downstream of ITGB8 (Fig. 7f,g). Furthermore, TRAP staining revealed that the knockdown of *Itgb8* enhanced osteoclastogenesis in OMD-treated BMMs (Fig. 7h,i). The knockdown of *Itgb8* in BMMs partially restores the osteoclastic activity induced by the absence of OMD, as indicated by TRAP staining following coculture of osteoblasts from *Omd*^flox/flox^ and *Omd*^*Δ*OC^ mice with wild-type BMMs (Supplementary Fig. 8a-c).

ITGB8 has been implicated in several downstream signaling pathways, including TGF-β, ERK, AKT and RhoA-YAP signaling^40–43^. In addition, *Rrm2* transcription is known to be regulated by JNK- and YAP/TEAD-dependent mechanisms^29,44^. We therefore examined the temporal activation status of these pathways following OMD treatment. WB analyses revealed that OMD stimulation did not markedly affect the phosphorylation of JNK, ERK, Smad2 or Smad3 at the examined time points, whereas p38 phosphorylation was suppressed at 120 min. By contrast, OMD robustly enhanced YAP phosphorylation at 30, 60 and 120 min and concomitantly reduced nuclear YAP localization (Fig. 7j,k and Supplementary Fig. 9a-c). Given the well-established role of ITGB8 in latent TGF-β activation, we further quantified TGF-β levels in culture supernatants by ELISA and observed no significant differences between the two groups (Supplementary Fig. 9d). Consistently, pharmacological inhibition of TGF-β signaling with SB-431542 failed to rescue the OMD-induced downregulation of *Rrm2* expression or osteoclast-related transcriptional markers (Supplementary Fig. 9e). Moreover, SB-431542 treatment did not restore the number of TRAP-positive osteoclasts (Supplementary Fig. 9f,g).

Because ITGB8 has been reported to promote YAP phosphorylation by suppressing RhoA-GTP activity^43^, we next assessed active RhoA following OMD exposure. OMD significantly reduced RhoA-GTP levels (Fig. 7l), indicating inhibition of RhoA activity downstream of ITGB8 activation. To further determine whether YAP/TEAD directly regulates *Rrm2* transcription, we performed ChIP assays to quantify TEAD occupancy at the *Rrm2* promoter^29^. OMD treatment markedly decreased the occupancy of the YAP-TEAD complex at the *Rrm2* promoter (Fig. 7m). In further support of ITGB8 dependence, ITGB8 knockdown effectively attenuated OMD-induced YAP phosphorylation (Fig. 7n and Supplementary Fig. 9h). Finally, to functionally probe the RhoA-YAP arm, we inhibited LATS1/2 using TRULI, thereby reducing inhibitory YAP phosphorylation and restoring nuclear YAP localization in OMD-treated cells (Supplementary Fig. 9i). Concurrently, TRULI treatment significantly increased the transcriptional levels of *Rrm2* and osteoclast-related differentiation markers in OMD-treated cells (Supplementary Fig. 9j). Consistently, the number of TRAP-positive osteoclasts was markedly increased upon TRULI treatment (Fig. 7o and Supplementary Fig. 9k).

Taken together, these data support an OMD-ITGB8-RhoA-YAP/TEAD axis that represses *Rrm2* transcription and slows osteoclastogenesis.

### Recombinant OMD and pharmacological inhibition of RRM2 partially attenuate bone loss induced by OVX or LPS

**Fig. 8:**
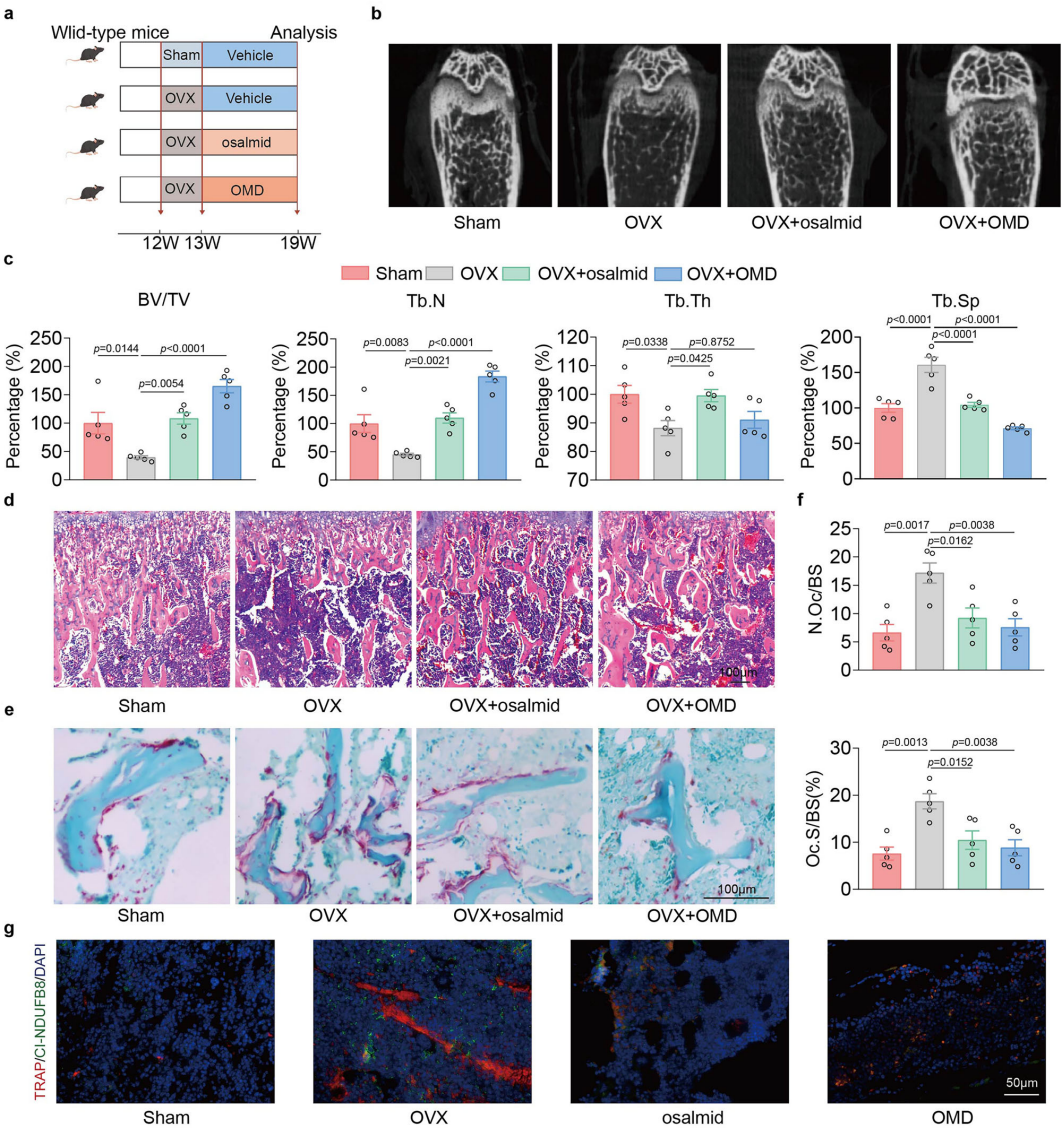
Both recombinant OMD protein and RRM2 inhibitor can mitigate OVX-induced bone loss. **a** Flowchart of the animal experiments. **b** Representative reconstructed 3D micro-CT images of the femurs in sham, OVX, OVX + osalmid, and OVX + OMD group mice (*n* = 5). **c** Quantification of BV/TV, Tb.N, Tb.Th and Tb.Sp from micro-CT images (*n* = 5). **d** H&E staining of the femurs from sham, OVX, OVX + osalmid, and OVX + OMD group mice (*n* = 5). Scale bar, 100 μm. **e**,**f** TRAP staining of the femurs from sham, OVX, OVX + osalmid, and OVX + OMD group mice (**e**) and quantification of N.Oc/BS and Oc.S/BS (%) (**f**) (*n* = 5). Scale bar, 100 μm. **g** Immunofluorescence of TRAP (red) and CI-NDUFB8 (green) in femur sections from sham, OVX, OVX + osalmid, and OVX + OMD group mice (*n* = 5). Scale bar, 50 μm. Data represent mean ± s.e.m. Experimental data for each quantitative analysis were replicated at least five times. Statistical significance was assessed using one-way ANOVA (**c** and **f**).

Given the critical roles of OMD and RRM2 in osteoclastogenesis, we investigated whether the administration of recombinant OMD protein or an RRM2 inhibitor (osalmid) to OVX mice could serve as an effective therapeutic intervention for osteoporosis (Fig. 8a). One week after inducing OVX, mice were administered osalmid at a dose of 100 mg/kg or recombinant OMD protein at a concentration of 200 ng/ml via intravenous injection three times per week. H&E staining demonstrated no significant changes in the liver, kidney and heart tissues of mice treated with OMD or osalmid compared with those of the other groups (Supplementary Fig. 10a). Micro-CT analysis revealed a significant increase in bone mass in mice treated with OMD and osalmid compared with that in OVX mice (Fig. 8b). Remarkably, in the OMD treatment group, BV/TV increased by 3.19-fold and Tb.N by 3.06-fold compared with the OVX group, while Tb.Sp, associated with osteoclastic activity, decreased by 56.6% (Fig. 8c). H&E staining revealed that treatment with OMD or osalmid improved the trabecular bone in the distal femur of OVX mice (Fig. 8d). TRAP staining showed that the number of osteoclasts on the trabecular surface of the femur in OMD- or osalmid-treated OVX mice was significantly lower than that in untreated OVX mice (Fig. 8e). The corresponding histological parameters, including N.Oc/BS and the percentage of Oc.S/BS (Oc.S/BS(%)), were also markedly reduced (Fig. 8f). In vivo immunofluorescence quantification within TRAP-positive osteoclasts showed that recombinant OMD or osalmid treatment reduced osteoclast abundance and was accompanied by a significant decrease in the NDUFB8 (complex I subunit) signal (colocalized with TRAP), consistent with reduced complex I/OXPHOS protein abundance in osteoclasts under these conditions (Fig. 8g).

We had shown that OMD and RRM2 inhibitors exhibit positive protective effects in chronic pathological bone loss, and thereafter, we further investigated whether they have therapeutic efficacy in acute bone resorption. To establish an inflammatory model of rapid bone loss, we administered intraperitoneal injections of 5 mg/kg LPS on days 0 and 4. Mouse femurs were then collected for analysis on day 8 (Supplementary Fig. 11a). Micro-CT analysis indicated that the application of recombinant OMD and RRM2 inhibitors can partially restore LPS-mediated rapid bone resorption, as indicated by a significant decrease in the osteoclast-related parameter Tb.Sp (Supplementary Fig. 11b,c). H&E and TRAP staining indicate that treatment with osalmid and OMD in LPS-treated mice rescues trabecular bone loss and the increased osteoclastogenesis caused by inflammatory bone resorption (Supplementary Fig. 11d-f). These in vivo data provide proof-of-concept evidence that OMD and RRM2 inhibitors can blunt excessive osteoclast activation and mitigate bone loss in both estrogen-deficiency and inflammatory settings.

## Discussion

An imbalance in bone remodeling is a key pathogenic mechanism of osteoporosis, with crosstalk between osteoblasts and osteoclasts tightly regulating bone remodeling^7^. While osteoblast-derived cues such as RANKL/OPG, chemokines and guidance molecules classically modulate osteoclastogenesis by shaping lineage commitment and signaling networks, our data support a distinct mechanism whereby an osteoblast-secreted SLRP engages integrin β8 to gate a metabolic checkpoint—RRM2-dependent mtDNA maintenance and mitochondrial ATP production—thereby constraining osteoclast differentiation. Previous research on OMD in the bone has primarily focused on its roles in osteogenesis, chondrogenesis and endochondral ossification, which involve both normal physiological processes^16^ and pathological conditions^24^. However, the role of OMD in osteoclasts remains unclear.

In this study, we explored the potential role of OMD in osteoclast-activated osteoporosis. We highlighted OMD’s role in the regulation of osteoclast formation in the context of osteoporosis. Our findings revealed that OMD levels negatively correlated with bone resorption markers. Both global and osteoblast-specific OMD-knockout mice exhibited increased osteoclast formation and subsequent bone loss. These bone phenotypes are inconsistent with previous reports^45^. We believe that conditional knockout mice can more accurately reflect gene function in specific cell types. Supporting evidence includes observations that Sirt1 global knockout mice exhibit no obvious liver defects, whereas hepatocyte-specific Sirt1-knockout mice develop fatty liver^46^. Similarly, compared with conventional knockout mice, mice with chondrocyte-specific conditional deletion of *Fgfr3* demonstrate more severe (and higher incidence of) chondrodysplasia-like lesions^47^. The tamoxifen-induced OMD knockout in adult mice used in our study may partially account for the discrepancy between the bone phenotype of our gKO mice and previously reported knockout models. Postnatal gene knockout probably circumvents potential confounding factors such as genetic redundancy, embryonic compensation, cellular plasticity during development, and developmental rewiring of gene networks^48–50^. Furthermore, phenotypic variations may also stem from differences in mouse inbreeding strains (‘genetic deviation’) and distinct knockout strategies used across studies.

It is worth mentioning that our analysis of single-cell sequencing data revealed a relatively low expression level of OMD in macrophages, and its expression exhibited minimal changes during osteoclast differentiation. This suggested that the osteoclast differentiation process might have a low dependency on OMD. To investigate whether endogenous OMD influenced osteoclast differentiation and murine bone mass, we generated *Omd*^ΔLysM^ mice. In vivo results demonstrated that the absence of OMD in osteoclast precursors did not lead to increased osteoclast activity or osteoporotic phenotypes. Similarly, a recent study reported that interleukin-9 (IL-9) secreted by T cells promotes the directed differentiation and functional maturation of germinal center B cells into memory B cells. However, B-cell-specific IL-9 deficiency did not impair the function of memory B cells^51^. A potential limitation of inducible CreERT2-based global deletion is that tamoxifen, a selective estrogen receptor modulator, can itself affect bone remodeling and may confound skeletal phenotyping, particularly when trabecular endpoints are assessed^35^. To minimize this concern, we treated *Omd*^flox^ littermate controls with the same tamoxifen/vehicle regimen and analyzed them in parallel; under our conditions, short-term tamoxifen exposure in *Omd*^flox^ mice did not significantly change trabecular bone parameters or osteoclast histomorphometry. Moreover, our central conclusion is supported by convergent evidence from osteoblast-lineage deletions and recombinant OMD gain-of-function experiments, which do not rely on tamoxifen induction.

Mechanistically, we identified an OMD-ITGB8-RhoA-YAP/TEAD-RRM2 axis as a previously unrecognized pathway through which an osteoblast-derived ECM cue restrains osteoclast differentiation. Integrins mediate both mechanical and chemical signals from the ECM by anchoring cells to the ECM, regulating cell survival and differentiation^52^. For example, integrin αvβ3, through interaction with the colony-stimulating factor 1 receptor, forms the cytoskeleton needed for osteoclast migration and activates the extracellular regulated protein kinases/c-Fos signaling pathway, regulating cell adhesion and differentiation. In addition, integrin α2β1 has been implicated in IL-7-induced osteoclast differentiation and inflammatory bone resorption^53^. Moreover, Liu et al. reported ITGB8 expression in mutants with impaired osteoclast formation^54^. Building on this framework, our work provides direct evidence that OMD binds ITGB8 on osteoclast precursors and thereby engages an ITGB8-dependent signaling route to suppress osteoclastogenesis, highlighting a previously underappreciated role for ITGB8 in bone metabolism.

The interplay between integrin signaling and other pathways, such as the wingless-related integration site and bone morphogenetic protein signaling pathways, ensures balanced bone remodeling by supporting proper osteoblast and osteoclast differentiation^55^. Given the established role of ITGB8 in latent TGF-β activation, we explicitly tested whether canonical TGF-β/Smad signaling contributes to the OMD phenotype. OMD did not alter Smad2/3 phosphorylation or active TGF-β levels in culture supernatants, and TGF-β receptor blockade (SB-431542) failed to rescue OMD-induced repression of *Rrm2* and osteoclastogenic markers, or TRAP-positive osteoclast formation. Together, these data indicate that OMD primarily signals through a TGF-β-independent ITGB8 pathway in osteoclast precursors under our experimental conditions.

Downstream of ITGB8, RRM2 emerged as a key mechanistic node. *Rrm2* was identified as one of the most differentially expressed genes in the RNA sequencing analysis between the OMD-treated and control groups, encoding a small subunit of ribonucleotide reductase, which is implicated in nucleotide metabolism and maintenance of the mitochondrial deoxynucleoside triphosphate pool^39^. Several studies have shown that RRM2 regulates mtDNA and mitochondrial proteins^56,57^. Our findings align with those of previous studies showing that RRM2 can regulate the expression of critical ETC functional proteins by influencing mtDNA copy number during osteoclast differentiation, mediating mitochondrial respiration and mitochondrial ATP production, and ultimately suppressing osteoclast differentiation. At the signaling level, our data further support an ITGB8-RhoA-YAP/TEAD axis linking extracellular OMD to transcriptional repression of *Rrm2*, as indicated by reduced RhoA-GTP, increased YAP phosphorylation with diminished nuclear YAP, decreased TEAD occupancy at the *Rrm2* promoter, and pharmacological reduction of YAP phosphorylation restored nuclear YAP, *Rrm2* expression and osteoclastogenesis. Because YAP lacks an intrinsic DNA-binding domain, TEAD occupancy provides a direct readout of YAP/TEAD transcriptional engagement at target loci. Future work will refine the promoter-level mechanisms (for example, TEAD-site mapping and promoter–reporter validation) and identify additional cofactors that may cooperate with YAP/TEAD in osteoclast precursors.

Osteoclast differentiation and bone resorption both require substantial amounts of energy, with mitochondrial ATP production serving as the primary energy source during the osteoclast differentiation process^58^. Previous studies indicated that blocking ETC from inhibiting OXPHOS energy production can reduce osteoclast formation^59^. In postmenopausal osteoporosis, estrogen signaling reduces OXPHOS activity and respiratory chain complex I subunit gene expression (*Ndufa7*, *Ndufb1*, *Ndufb2* and *Ndufs8*) to inhibit osteoclast differentiation^58^. CTSK, a key functional protein in osteoclasts, is closely associated with acetyl-CoA produced in the tricarboxylic acid cycle^37^. Notably, CTSK expression significantly decreases when mitochondrial respiration is inhibited^37^. Furthermore, a substantial body of research has elucidated the influence of the SLRP family on mitochondrial function. For instance, decorin can polarize decidual macrophages to the M1 phenotype by modulating mitochondrial metabolism^60^, while simultaneously inducing VEGFR2-dependent mitochondrial fragmentation and loss of mitochondrial membrane potential in endothelial cells^61^. In our study, the convergence of respiration, ATP rate, membrane potential and untargeted metabolomics data support a model in which OMD preferentially impairs mitochondrial oxidative metabolism during osteoclastogenesis. OMD suppressed mtDNA copy number, the expression of ETC functional proteins, mitochondrial respiration and ATP production, thereby inhibiting osteoclast differentiation. This study introduces an exogenous protein-mediated regulation of osteoclast differentiation by modulating osteoclast energy metabolism.

Osteoclastogenesis requires substantial metabolic adaptation. To evaluate whether OMD induces broader metabolic remodeling beyond bioenergetic readouts, we performed untargeted metabolomics in osteoclast precursors. Unsupervised global profiling revealed a clear separation between control and OMD-treated samples, supporting a broad shift in the cellular metabolome under OMD stimulation. Among the most prominent changes, we observed coordinated alterations in acylcarnitine species, nucleotide-related metabolites (including AMP/adenosine-related features) and redox-associated metabolites (notably GSSG). These steady-state signatures are directionally consistent with a mitochondrial energy-stress state and perturbed nucleotide/redox homeostasis under the OMD condition. Importantly, these metabolomics findings converge with our functional measurements showing reduced mitochondrial respiration and mitochondrial ATP production (OCR and ATP-rate assays) and with the transcriptomic/mechanistic identification of the RRM2-mtDNA axis, collectively supporting a model in which OMD limits mitochondrial ATP production during osteoclastogenesis.

Of note, untargeted metabolomics captures relative metabolite abundances and therefore provides a snapshot rather than direct quantification of pathway flux. Stable isotope tracing approaches (for example, ^13^C-glucose) will be valuable in future work to delineate substrate utilization and TCA cycle flux under OMD stimulation. Nonetheless, the concordance across respiration, ATP rate, mitochondrial membrane potential and untargeted metabolomics datasets provides global support for metabolic remodeling and supports reduced mitochondrial ATP production in OMD-treated osteoclast precursors.

OMD supplementation inhibits osteoclastogenesis and delays bone loss. A previous study demonstrated that OMD promotes the osteogenic differentiation of human dental pulp stem cells and bone formation^62^. We also validated the positive effects of OMD on osteogenic differentiation of bone marrow mesenchymal stem cells in vitro (Supplementary Fig. 12a,b). Thus, OMD, a target that promotes osteogenesis and inhibits osteoclastogenesis, has broad prospects for the prevention and treatment of osteoporosis, especially in the context of osteoporotic fractures. Although recombinant OMD administration ameliorated bone loss in OVX- and LPS-induced mouse models, these experiments should be interpreted as proof of concept. The pharmacological translation of OMD will require formal characterization of stability, bioavailability, tissue distribution, immunogenicity and long-term safety, as well as comparative efficacy studies against established antiresorptive and anabolic agents. Rather than positioning OMD as superior to existing therapies, our findings provide mechanistic insight into an osteoblast-to-osteoclast metabolic communication axis and nominate components of the ITGB8-YAP/TEAD-RRM2 pathway as potential targets for future antiresorptive strategies, including rational combination approaches.

In conclusion, our study identifies osteoblast-derived OMD as a matrix-associated regulator that restrains osteoclastogenesis. By engaging an ITGB8-RhoA-YAP/TEAD-RRM2 axis in osteoclast precursors, OMD reduces mitochondrial respiration and mitochondrial ATP production during osteoclastogenesis, with only limited glycolytic compensation. These findings connect osteoblast-derived extracellular signals to bioenergetic control of osteoclast differentiation and provide a mechanistic framework for future studies exploring OMD-centered pathways in bone remodeling.

## Supplementary information

Supplementary Information

## Data Availability

The datasets used and/or analyzed during the current study are available from the corresponding author on reasonable request.
