# Supplementary material for: Osteoblast-derived osteomodulin restrains osteoclastogenesis via ITGB8/RRM2-mediated reduction of mitochondrial respiration and mitochondrial ATP production
Source: Exp Mol Med. 2026 Mar 12;58(3):879–97. doi: 10.1038/s12276-026-01682-7 (PMC13049008; doi:10.1038/s12276-026-01682-7)
Supplement: Supplementary file 1 — Supplementary Information [file 12276_2026_1682_MOESM1_ESM.pdf]

Supplementary Materials for

Osteoblast-derived osteomodulin restrains osteoclastogenesis *via* ITGB8/RRM2-mediated reduction of mitochondrial respiration and mitochondrial ATP production

Xiaowen Jiang *et al.*

✉ ✉ Corresponding author: Xiaohua Yu, Email: Xiaohua.yu@zju.edu.cn; Weixu Li, Email: zrlwx@zju.edu.cn; Wei Zhang, Email: zhangweilook@zju.edu.cn

This PDF file includes:

Supplementary Fig. 1 to 12
Supplementary Table 1 to 2

Supplementary Text

Supplementary Fig. 1. Correlation analysis between OMD and bone metabolism indicators in serum. (**a**) Correlation analysis of OMD levels in female serum with Ca^{2+} (n=36). (**b**) Correlation analysis of OMD levels in mice serum with β-C-terminal telopeptide of type I collagen (β-CTX) (n=26). By *Pearson*'s correlation analysis (**a** and **b**).

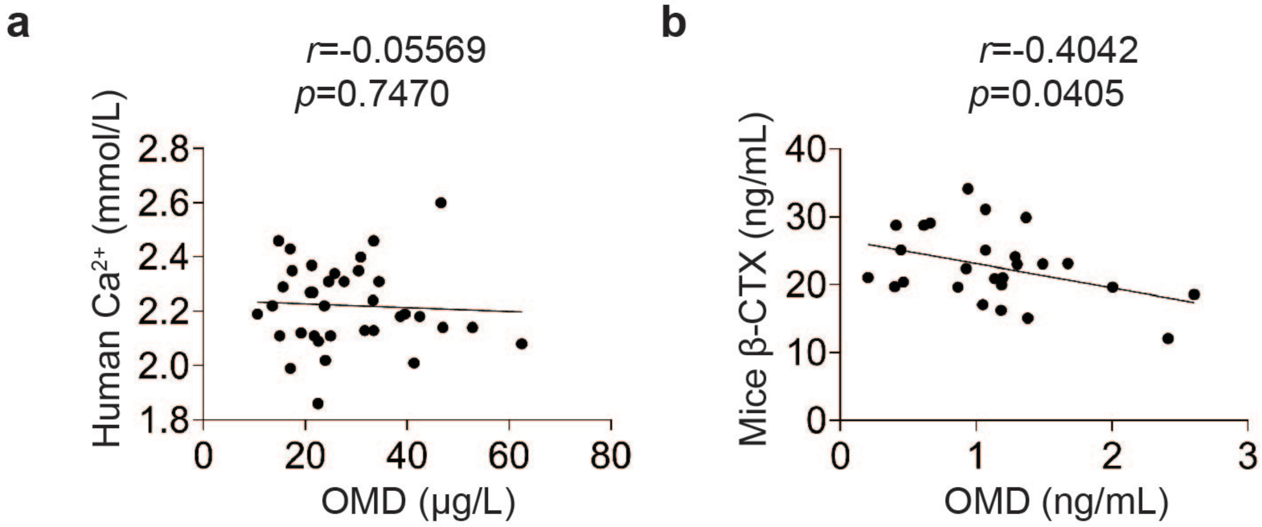

Supplementary Fig. 2. Identification of OMD Knockout Mice. (**a**) Cre-mediated recombination eliminates exon 3 of the *Omd* gene. (**b**) Identification of representative DNA agarose gel electrophoresis images for *Omd*^{flox} and *Omd*$^{\Delta CAG}$ (administered corn oil or tamoxifen). (**c**) Representative immunofluorescent images showing OMD (green) expression in femoral bone sections from *Omd*^{flox} and *Omd*$^{\Delta CAG}$ mice administered corn oil or tamoxifen, 100 µm. (**d**) Identification of representative DNA agarose gel electrophoresis images for *Omd*^{flox} and *Omd*$^{\Delta LysM}$. (**e**) Representative immunofluorescence staining of OMD (green) in osteoclast precursors of *Omd*^{flox} and *Omd*$^{\Delta LysM}$ mice. Scale bar, 50 µm. (**f**) Identification of representative DNA agarose gel electrophoresis images for *Omd*^{flox} and *Omd*$^{\Delta OC}$. (**g**) Representative immunofluorescence staining of OMD (green) in femoral sections of *Omd*^{flox} and *Omd*$^{\Delta OC}$ mice. Scale bar, 100 µm. (**h**) Identification of representative DNA agarose gel electrophoresis images for *Omd*^{flox} and *Omd*$^{\Delta DMP1}$. (**i**) Representative immunofluorescence staining of OMD (green) in femoral sections of *Omd*^{flox} and *Omd*$^{\Delta DMP1}$ mice. Scale bar, 100 µm.

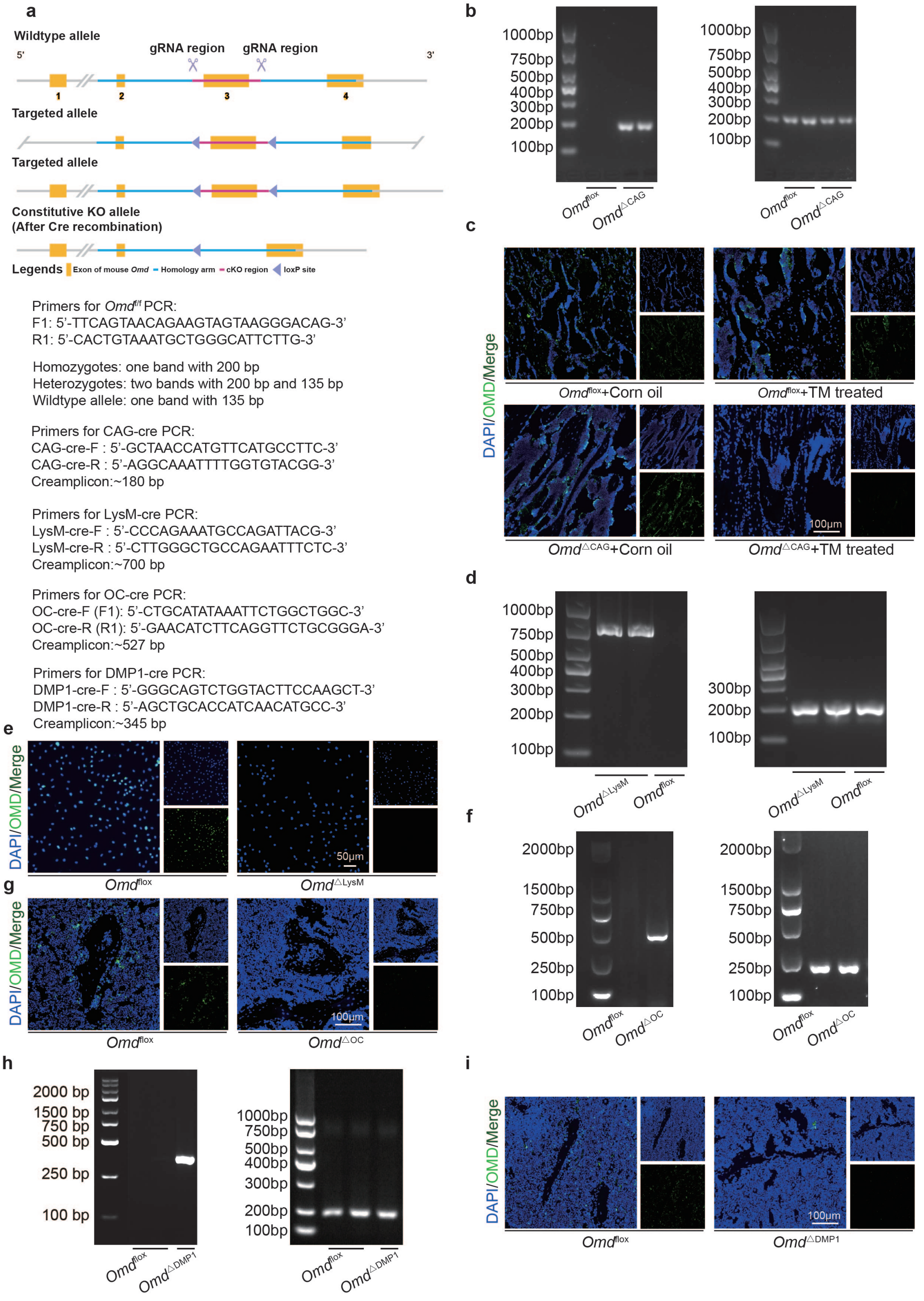

Supplementary Fig. 3. The absence of OMD in osteoclast precursor cells produces no overt skeletal phenotype. (**a**) H&E staining of the femurs from 3-month-old *Omd*^{flox} and *Omd*$^{\Delta LysM}$ mice (n=5). Scale bar, 100 µm. (**b**) TRAP staining of the femurs from 3-month-old *Omd*^{flox} and *Omd*$^{\Delta LysM}$ mice (n=5). Scale bar, 100 µm. (**c**) Quantification of N.Oc/BS and Oc.S/BS(%) (n=5) (**d**) Protein levels of CTSK and OMD were assessed by Western blotting after 2 and 4 days of induction (n=3). (**e**) Quantitative analysis of OMD protein during the differentiation of BMMs (n=3). (**f**) Statistical analysis of *Omd* mRNA expression during osteoclast differentiation in the GSE176265 dataset was performed (n=6). (**g**) The tSNE plot illustrates the distribution of the *Omd* gene. Data represent mean ± SEM. Experimental data for each quantitative analysis were replicated at least three times. By one-way *ANOVA* (**e** and **f**) or two-way *ANOVA* (**c**).

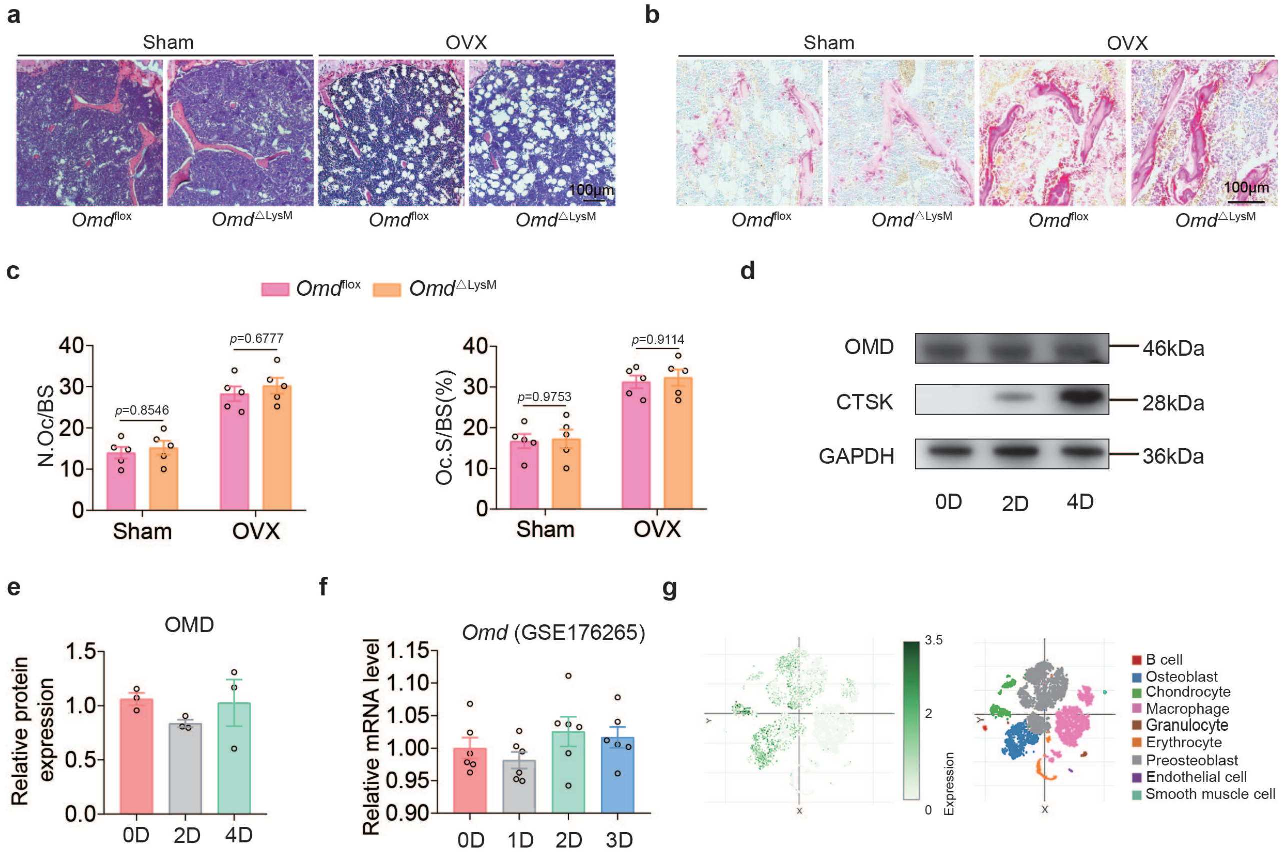

Supplementary Fig. 4. Deletion of the *Omd* gene in osteoblasts leads to bone loss and increased osteoclast activity. (**a**) Representative reconstructed 3D micro-CT images of the femurs of 3-month-old *Omd*^{flox} and *Omd*$^{\Delta DMP1}$ mice (n=5). (**b**) Quantification of BV/TV, Tb.N, Tb.Th and Tb.Sp from micro-CT images (n=5). (**c**) H&E staining of the femurs from 3-month-old *Omd*^{flox} and *Omd*$^{\Delta DMP1}$ mice (n=5). Scale bar, 100 μm. (**d**) TRAP staining of the femurs from 3-month-old *Omd*^{flox} and *Omd*$^{\Delta DMP1}$ mice (n=5). Scale bar, 100 μm. (**e**) Quantification of N.Oc/BS (osteoclast number/bone surface) and the percentage of osteoclast surface per bone surface (Oc.S/BS(%)) (n=5). Data represent mean ± SEM. Experimental data for each quantitative analysis were replicated at least three times. By unpaired *t* test (**b** and **e**).

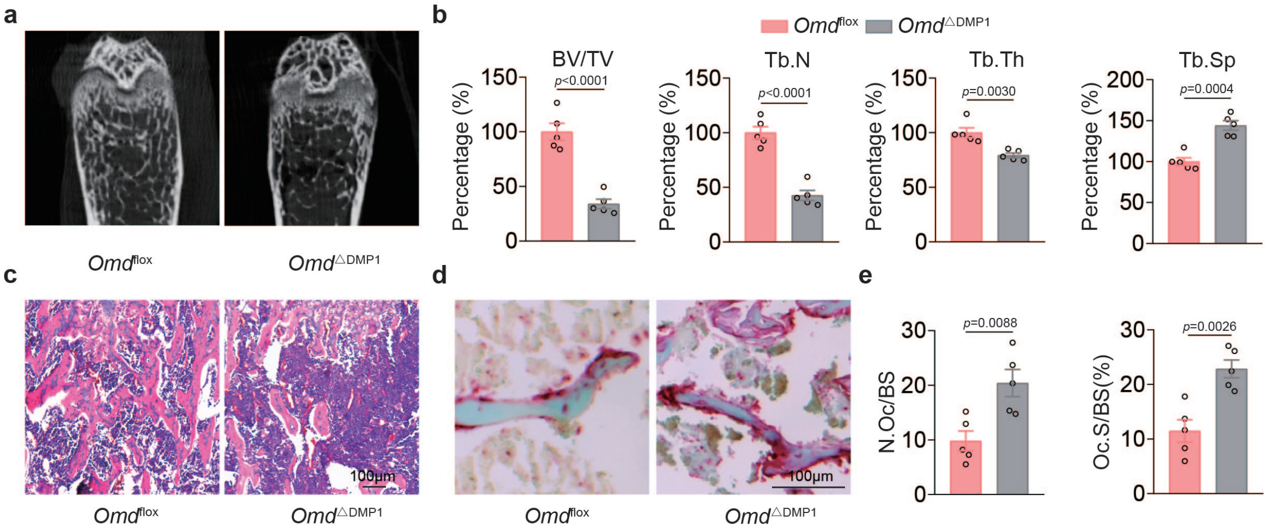

Supplementary Fig. 5. OMD protein inhibits osteoclast differentiation *in vitro*. (**a** and **b**) The mRNA levels of osteoclastogenesis-related genes were assessed via qRT-PCR after after three days of co-culture (n=3). (**c** and **d**) TRAP staining was performed on the control group and the group treated with 100 ng/mL recombinant OMD protein after 2 and 4 days of treatment with 30 ng/mL M-CSF and 100 ng/mL RANKL and quantification of nuclei numbers of TRAP-positive multinuclear cells (n=3). Scale bar, 100 μm. (**e**) Quantification of the percentage of positive area in F-actin ring staining results and the number of nuclei per osteoclast was performed (n=3). (**f**) The mRNA levels of osteoclastogenesis-related genes were assessed via qRT-PCR following a 1-d treatment with LPS and various concentrations of OMD. (n=3). Data represent mean ± SEM. Experimental data for each quantitative analysis were replicated at least three times. By unpaired *t* test (**a** and **b**) or two-way *ANOVA* (**d, e** and **f**).

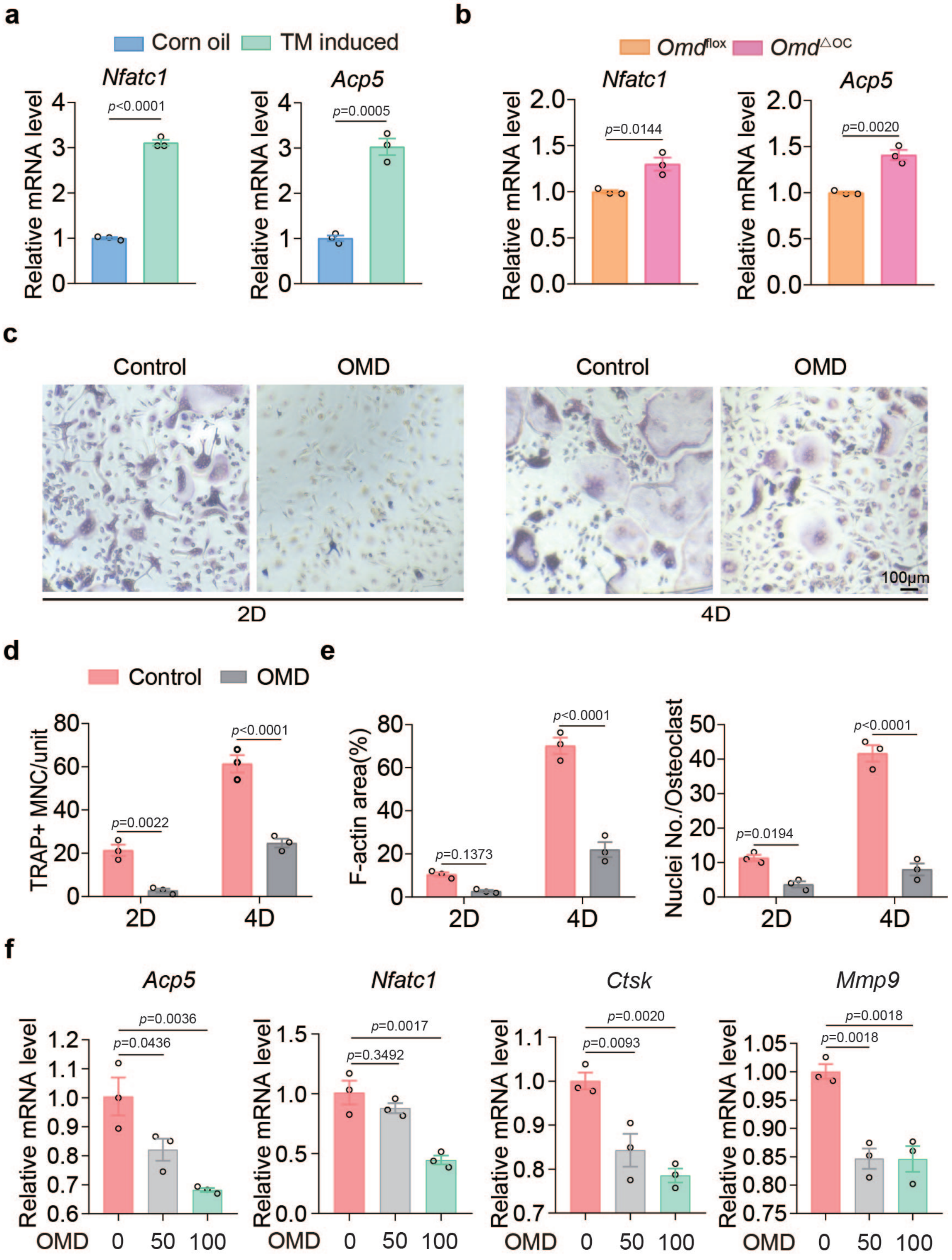

Supplementary Fig. 6. OMD inhibits osteoclast differentiation through RRM2-mediated mitochondrial respiration and mitochondrial ATP production. (**a**) The heatmap illustrated the significantly differential genes in the OXPHOS pathway between the control group and the OMD-treated group. Red denotes upregulation, while blue signifies downregulation (n=3). (**b**) Immunofluorescence of TRAP (red) and CI-NDUFBB (green) in femur sections from *Omd*^{flox} and *Omd*$^{\Delta\text{OC}}$ mice (n=5). Scale bar, 50 µm. (**c**) Representative 2-NBDG fluorescence images showing glucose uptake in control and OMD-treated group. (n=3). Scale bar, 50 µm. (**d**) Representative TMRE fluorescence images showing mitochondrial membrane potential in control and OMD-treated group. (n=3). Scale bar, 50 µm. (**e**) Principal component analysis (PCA) of metabolomic profiles from control and OMD-treated groups (n=5). (**f**) Heatmap showing representative differentially abundant metabolites related to energy metabolism in OMD-treated versus control groups (n=5).

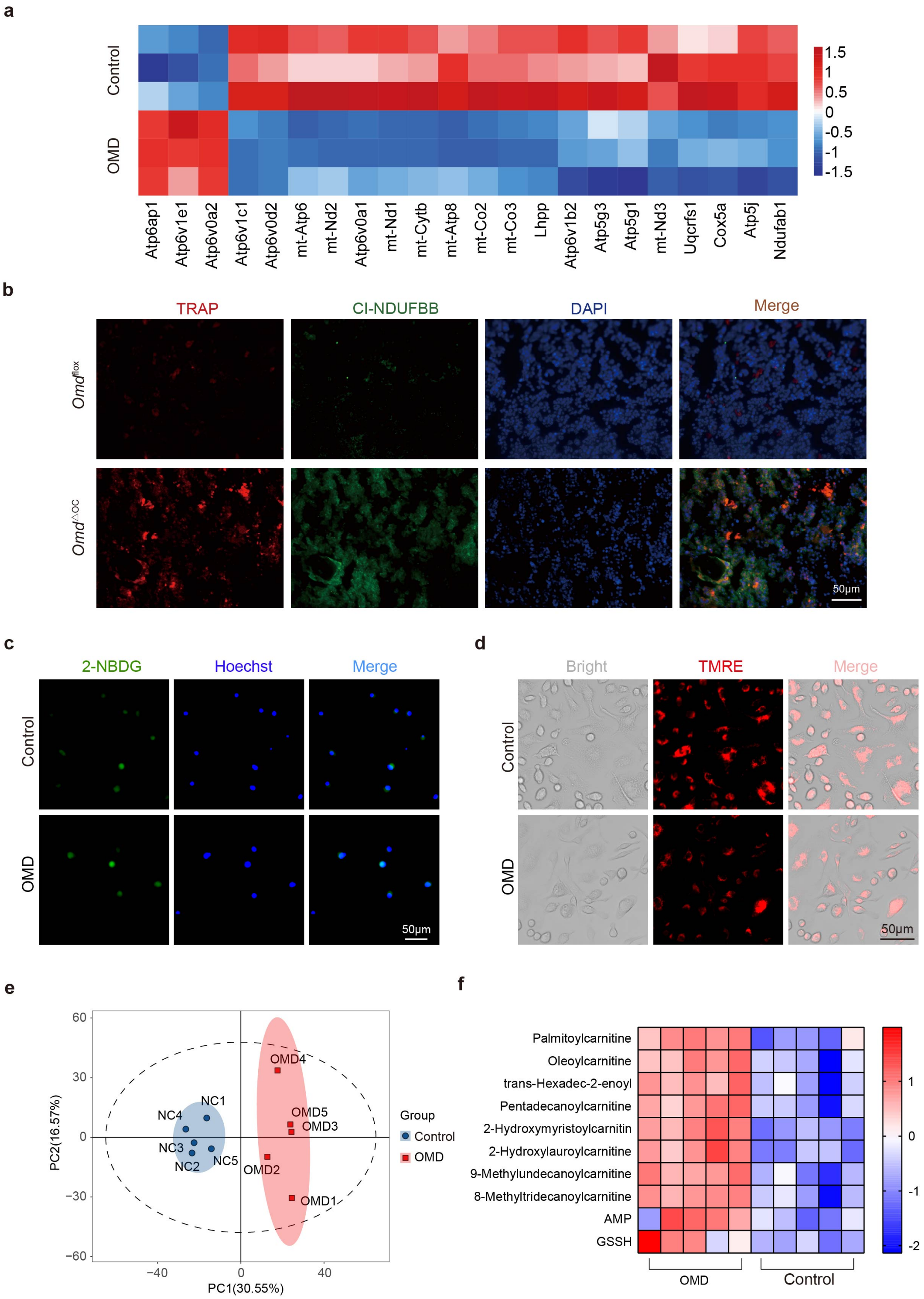

Supplementary Fig. 7. RRM2 restoration rescues OMD-induced mitochondrial respiration and mitochondrial ATP production during osteoclast differentiation. (**a**) Metabolic pathway enrichment analysis indicated that nucleotide metabolism was significantly different between the control group and the OMD-treated group. (**b**) The heatmap revealed significant differences in nucleotide-related metabolites between the control group and the OMD-treated group. (**c**) Immunofluorescence of TRAP (red) and RRM2 (green) in femur sections from *Omd*^{flox} and *Omd*$^{\Delta OC}$ mice (n=5). Scale bar, 50 µm. (**d**) Representative 2-NBDG fluorescence images showing glucose uptake in different groups. (n=3). Scale bar, 50 µm. (**e**) Representative TMRE fluorescence images showing mitochondrial membrane potential in different groups. (n=3).

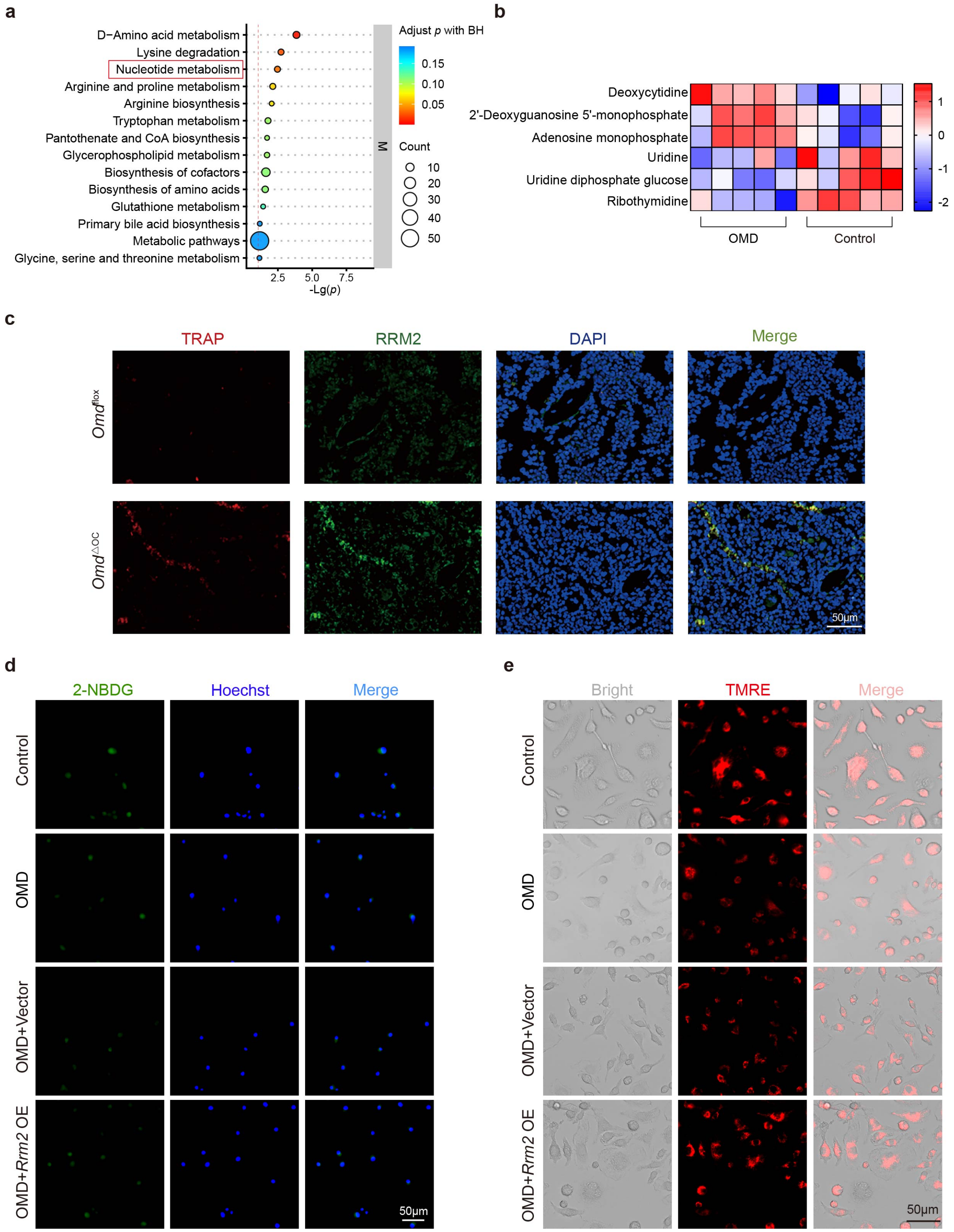

Supplementary Fig. 8. ITGB8 serves as the functional receptor in OMD-mediated osteoblast-osteoclast communication. (**a**) A schematic representation of co-cultivation. (**b** and **c**) TRAP staining was performed on osteoclasts after 9 days of co-culture, followed by a quantitative analysis of the nuclei counts in TRAP-positive multinuclear cells (n=3). Scale bar, 100 μm. Data represent mean ± SEM. Experimental data for each quantitative analysis were replicated at least three times. By one-way *ANOVA* (**c**).

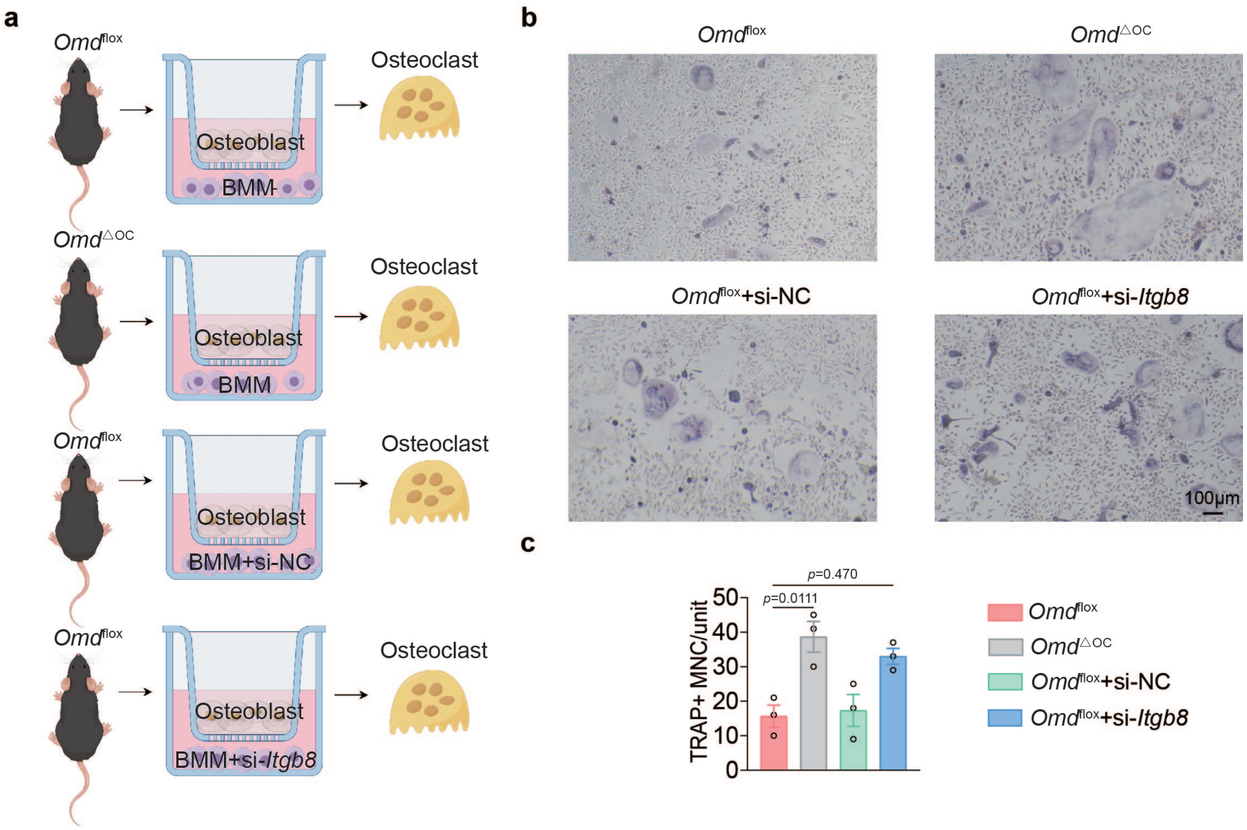

Supplementary Fig. 9. OMD suppresses osteoclastogenesis through an ITGB8–RhoA–YAP-RRM2 axis independently of TGF-β signaling. (**a** and **b**) Quantitative analysis of phosphorylation levels of indicated proteins at indicated time (0, 30, 60 and 120 min) between the control group and the OMD-treated group (n=3). (**c**) Representative immunofluorescence images showing nuclear localization of YAP in OMD treated or control groups. (**d**) TGF-β levels in the culture medium of control and OMD-treated cells, as measured by ELISA (n=3). (**e**) qRT-PCR analysis of *Rrm2* and osteoclast differentiation marker expression in the indicated treatment groups (n=3). (**f** and **g**) After a 4-day induction, TRAP staining was conducted on BMMs exposed to different treatments, followed by a quantitative analysis of the nuclei counts in TRAP-positive multinuclear cells (n=3). Scale bar, 100 μm. (**h**) Quantitative analysis of phosphorylation levels of YAP (n=3). (**i**) Representative immunofluorescence images showing nuclear localization of YAP in different treatment groups. (**j**) qRT-PCR analysis of *Rrm2* and osteoclast differentiation marker expression in the indicated treatment groups (n=3). (**k**) Quantitative analysis of the nuclei counts in TRAP-positive multinuclear cells (n=3). Data represent mean ± SEM. Experimental data for each quantitative analysis were replicated at least five times. By unpaired *t* test (**a, b** and **d**) or one-way *ANOVA* (**e, g, h, j** and **k**).

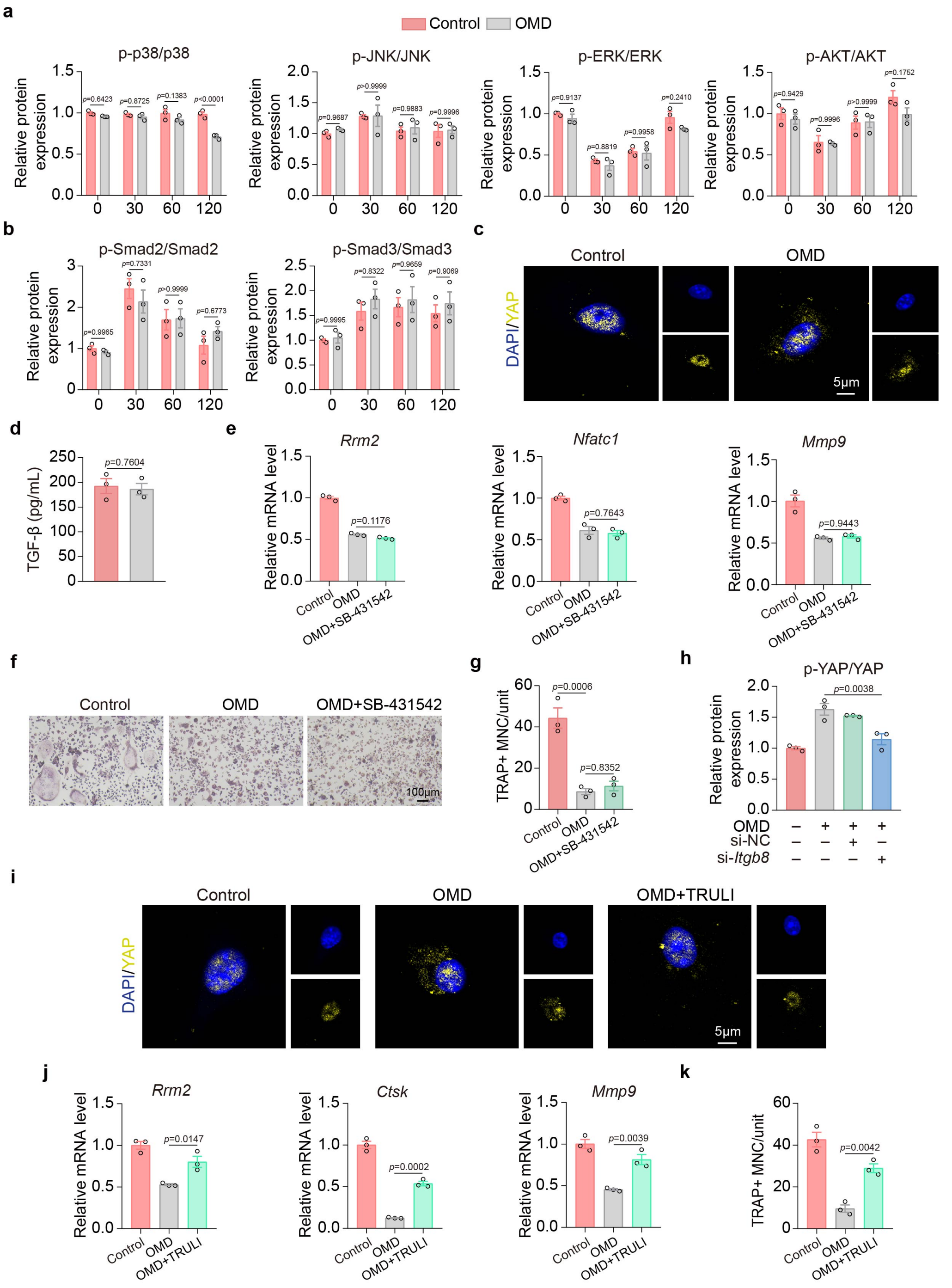

Supplementary Fig. 10. Recombinant OMD protein and osalmid exhibit no significant toxicity *in vivo*. (**a**) H&E staining of liver, kidney and heart from Sham, OVX, OVX+osalmid, OVX+OMD group mice (n=5). Scale bar, 50 μm.

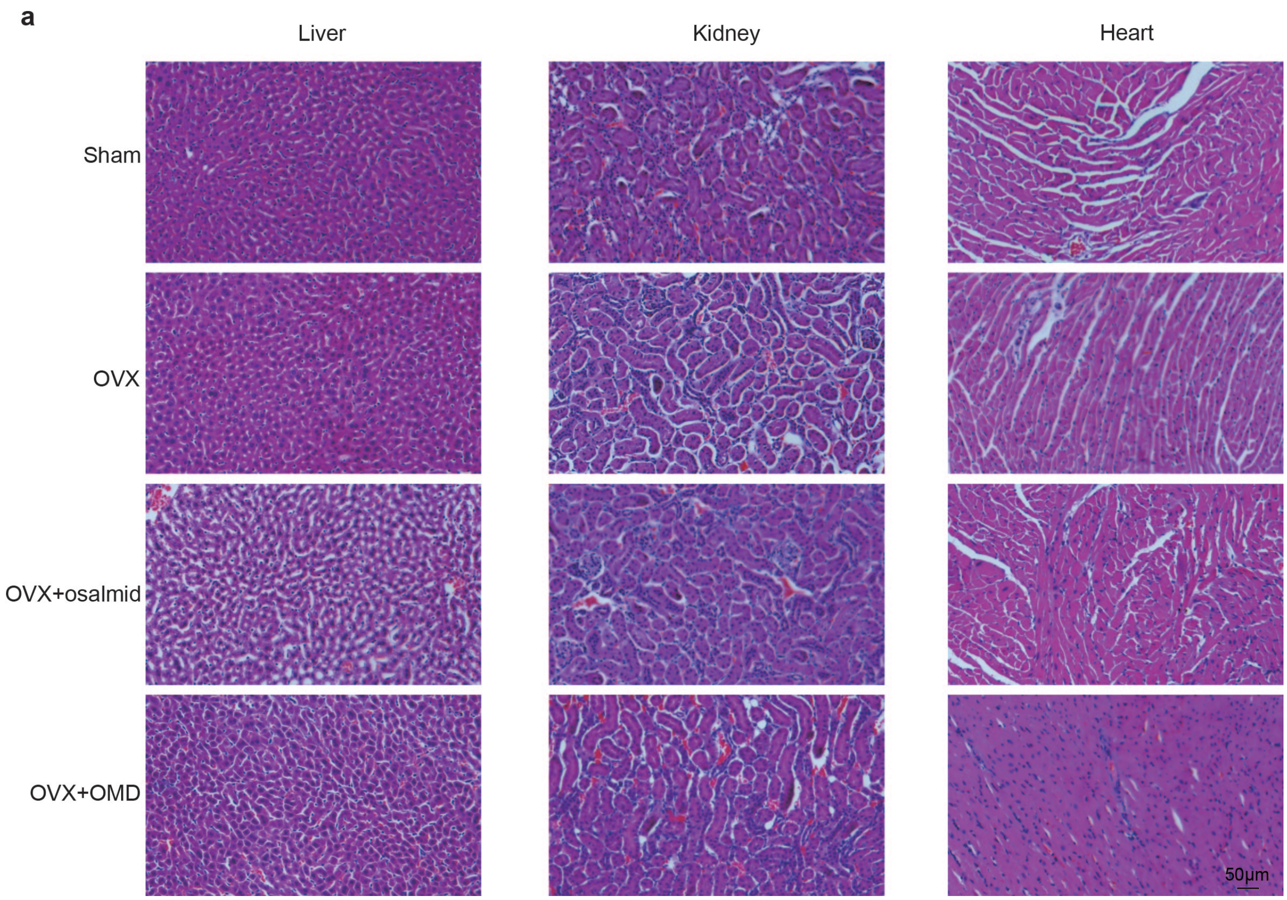

Supplementary Fig. 11. Both recombinant OMD protein and RRM2 inhibitor can mitigate LPS-induced bone loss. (**a**) Flowchart of the animal experiments. (**b**) Representative reconstructed 3D micro-CT images of the femurs in Sham, LPS, LPS+osalmid, LPS+OMD group mice (n=5). (**c**) Quantification of BV/TV, Tb.N, Tb.Th and Tb.Sp from mirco-CT images (n=5). (**d**) H&E staining of the femurs from Sham, LPS, LPS+osalmid, LPS+OMD group mice (n=5). Scale bar, 100 μm. (**e** and **f**) TRAP staining of the femurs from Sham, LPS, LPS+osalmid, LPS+OMD group mice and quantification of N.Oc/BS (osteoclast number/bone surface) and the percentage of osteoclast surface per bone surface (Oc.S/BS(%)) (n=5). Scale bar, 100 μm. Data represent mean ± SEM. Experimental data for each quantitative analysis were replicated at least five times. By one-way *ANOVA* (**c** and **f**).

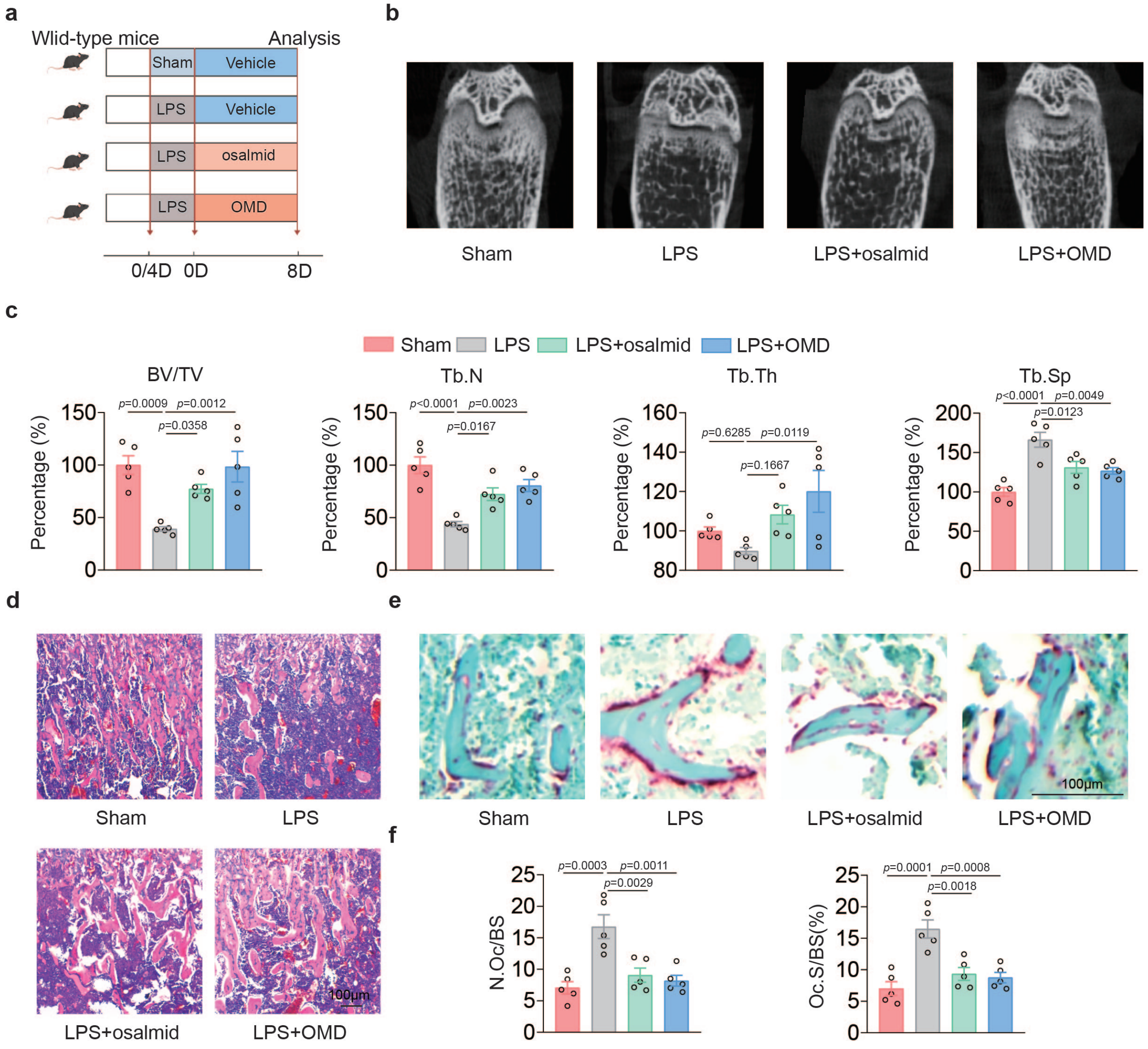

Supplementary Fig. 12. OMD promotes the osteogenic differentiation of BMSCs *in vitro*. (**a**) After a 3-day treatment of BMSCs with various concentrations of OMD under osteogenic induction, protein levels of COL-1 and RUNX2 were assessed by Western blotting (n=3). (**b**) Quantitative analysis of COL-1 and RUNX2 protein (n=3). By one-way *ANOVA* (**b**).

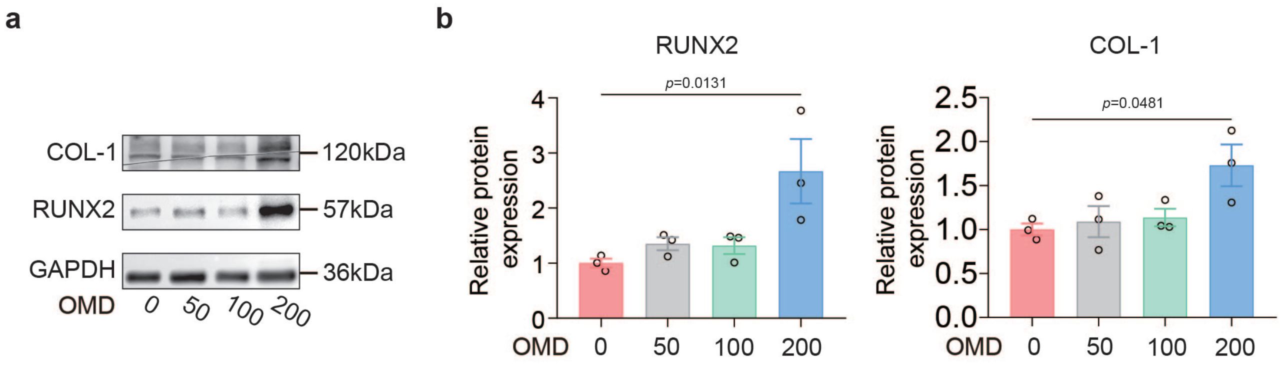

Supplementary Table 1. Characteristics of the study participants

Group	Control	Osteoporosis	All age groups
Age (years), mean (s.d.)	68.4, 9.8	72.5, 9.5	56.6, 13.6
Sample size	14	15	36
Sex	Female	Female	Female

Control group age vs. Osteoporosis group age: *p*= 0.2888 (unpaired *t* test)

Supplementary Table 2. Primer sequences for RT-qPCR, PCR, ChIP-qPCR and si-RNA

Gene Name-species	Forward (5'→3')	Reverse (5'→3')
β-actin-M	CATTGCTGACAGGATGCAGAAGG	TGCTGGAAGGTGGACAGTGAGG
Rrm2-M	CTCAAGAAACGGGGGCTGAT	CTCTGCTGGCTTGTGTACCA
Itgb3-M	AAACTCGCCAGGTGGTATGTGA	CTGGTGCACACTGAAACGAAGA
Mmp9-M	CAAAGACCTGAAAACCTCCAA	GGTACAAGTATGCCTCTGCCA
Nfatc1-M	CCGTTGCTTCCAGAAAATAACA	TGTGGGATGTGAACTCGGAA
Ctsk-M	ACTGCCTTCCAATACGTGCA	CCCTCTGCATTTAGCTGCCT
Acp5-M	CACTCCCACCCTGAGATTTGT	CCCCAGAGACATGATGAAGTCA
Cytb-M	TGCATACGCCATTCTACGCT	AGGCTTCGTTGCTTTGAGGT
Cox3-M	CGGAAGTATTTTCTTTGCAGGAT	CAGCAGCCTCCTAGATCATGTG
Cox1-M	TTTTCAGGCTTCACCCTAGATGA	GAAGAATGTTATGTTTACTCCTACGAA
Nd2-M	CATCACTCTATTCTGCCTAGCAA	TCCTCGGGCCATGATTATAGTAC
βglobin-M	GAAGCGATTCTAGGGAGCAG	GGAGCAGCAGCGATTCTGAGTAGA
mtCO2-M	CCGACTAAATCAAGCAACA	CAATGGGCATAAAGCTATGG
Itgb8-M	TCCAGCCTTGGTTCTTGG	CACATTTGCAGAGCCACATC
CAG-ERT2-cre-M	GCTAACCATGTTCATGCCTTC	AGGCAAATTTTGGTGTACGG
LysM-cre-M	CCCAGAAATGCCAGATTACG	CTTGGGCTGCCAGAATTTCTC
OC-cre-M	CTGCATATAAATTCTGGCTGGC	GAACATCTTCAGGTTCTGCGGGA
DMP1-cre-M	GGGCAGTCTGGTACTTCCAAGCT	AGCTGCACCATCAACATGCC
Omd-flox-M	TTCAGTAACAGAAGTAGTAAGGGACAG	CACTGTAAATGCTGGGCATTCTTG
Rrm2 (ChIP)	CGGATAACGGAGGAATGGAA	TCTTCCATGCTAAGCTAACTG
Itgb8-siRNA-M	CAUUAGACAAGUGAUACUA	UAGUAUCACUUGUCUAAUG
